# The use and impact of surveillance-based technology initiatives in inpatient and acute mental health settings: a systematic review

**DOI:** 10.1186/s12916-024-03673-9

**Published:** 2024-11-29

**Authors:** Jessica L. Griffiths, Katherine R. K. Saunders, Una Foye, Anna Greenburgh, Ciara Regan, Ruth E. Cooper, Rose Powell, Ellen Thomas, Geoff Brennan, Antonio Rojas-García, Brynmor Lloyd-Evans, Sonia Johnson, Alan Simpson

**Affiliations:** 1 Department of Health Service and Population Research (HSPR), NIHR Policy Research Unit in Mental Health (MHPRU), Institute of Psychiatry, Psychology & Neuroscience, King’s College London, London, UK; 2 NIHR Policy Research Unit in Mental Health (MHPRU), Division of Psychiatry, University College London, London, UK; 3 Department of Health Service and Population Research (HSPR), Institute of Psychiatry, Psychology & Neuroscience, King’s College London, London, UK; 4 Florence Nightingale Faculty of Nursing, Midwifery and Palliative Care, King’s College London, London, UK; 5 School of Geography, Politics, and Sociology, Newcastle University, Newcastle Upon Tyne, UK; 6 London, UK; 7 Department of Behavioural Science Methodology, Faculty of Psychology, University of Granada, Granada, Spain; 8 Camden and Islington NHS Foundation Trust, London, UK

**Keywords:** Surveillance technology, Inpatient, Mental health, Vision-based patient monitoring and management, Body-worn cameras, CCTV, Video monitoring, Security, Safety management

## Abstract

**Background:**

The use of surveillance technologies is becoming increasingly common in inpatient mental health settings, commonly justified as efforts to improve safety and cost-effectiveness. However, their use has been questioned in light of limited research conducted and the sensitivities, ethical concerns and potential harms of surveillance. This systematic review aims to (1) map how surveillance technologies have been employed in inpatient mental health settings, (2) explore how they are experienced by patients, staff and carers and (3) examine evidence regarding their impact.

**Methods:**

We searched five academic databases (Embase, MEDLINE, PsycInfo, PubMed and Scopus), one grey literature database (HMIC) and two pre-print servers (medRxiv and PsyArXiv) to identify relevant papers published up to 19/09/2024. We also conducted backwards and forwards citation tracking and contacted experts to identify relevant literature. The Mixed Methods Appraisal Tool assessed quality. Data were synthesised narratively.

**Results:**

Thirty-two studies met the inclusion criteria. They reported on CCTV/video monitoring (*n* = 13), Vision-Based Patient Monitoring and Management (*n* = 9), body-worn cameras (*n* = 6), GPS electronic monitoring (*n* = 2) and wearable sensors (*n* = 2). Sixteen papers (50.0%) were low quality, five (15.6%) medium quality and eleven (34.4%) high quality. Nine studies (28.1%) declared a conflict of interest. Qualitative findings indicate patient, staff and carer views of surveillance technologies are mixed and complex. Quantitative findings regarding the impact of surveillance on outcomes such as self-harm, violence, aggression, care quality and cost-effectiveness were inconsistent or weak.

**Conclusions:**

There is currently insufficient evidence to suggest that surveillance technologies in inpatient mental health settings are achieving their intended outcomes, such as improving safety and reducing costs. The studies were generally of low methodological quality, lacked lived experience involvement, and a substantial proportion (28.1%) declared conflicts of interest. Further independent coproduced research is needed to more comprehensively evaluate the impact of surveillance technologies in inpatient settings. If they are to be implemented, all key stakeholders should be engaged in the development of policies, procedures and best practice guidance to regulate their use, prioritising patients’ perspectives.

**Supplementary Information:**

The online version contains supplementary material available at 10.1186/s12916-024-03673-9.

## Background

Inpatient mental health settings are challenging environments, both for those receiving and those delivering mental healthcare. The core purpose of inpatient wards is to provide a physically and psychologically safe place for people experiencing acute mental health difficulties to recover and receive care; however, both patients and staff have reported feeling unsafe on wards [1–3]. Inpatient mental health patients report (re)traumatising experiences including abuse, coercion, aggression and violence on wards from staff and/or other patients [4–8]. Staff also report abuse and violence on the wards from patients [9, 10], as well as having to risk-assess for and respond to incidents of self-harm and suicide attempts, which are prevalent in these settings [11].

In this context, some mental health service providers in the UK are increasing their use of surveillance-based technologies in inpatient settings [12]. Such surveillance technologies include closed circuit television (CCTV), body-worn cameras (BWCs) and remote monitoring devices (such as smart watches, Global Positioning System (GPS) trackers and infrared cameras). Use of these technologies is justified on the basis that they may be able to detect or prevent aggressive and violent incidents, reduce self-harm incidents and suicide attempts, improve staff and patient safety, change patient behaviour and staff conduct, provide accurate records to help resolve complaints and to contribute to legal cases, and reduce staffing costs [13–17]. Conflict and providing adequate staffing on wards are costly [18] but interrelated [18, 19]. Reducing cost is a driving force for many service providers; therefore, surveillance technologies may appear to offer a cost-effective solution.

The use of video technologies implemented with the stated purpose of improving security is becoming increasingly common. For example, in the UK, BWCs are now used by the police [20], emergency healthcare workers including paramedics [21–23], and retail staff [24–26]. However, the use of some of these technologies on inpatient wards is controversial [27, 28]. Patient and service user groups, as well as advocates and disability rights activists, have consistently called for scrutiny of these technologies regarding potential risks of iatrogenic harm and ethical concerns [29, 30]. For example, issues raised by the Stop Oxevision campaign [31] include (i) ethical considerations around use of surveillance technologies and obtaining informed consent (for example, concerns about the ability of services to provide adequate information for informed consent, potential consequences for patients not providing or withdrawing consent, and whether consent can reasonably be given to being filmed or recorded whilst acutely unwell on an inpatient ward), (ii) concerns about data access, storage, security and human rights violations, (iii) distress caused by being recorded or monitored, or the exacerbation of existing paranoia, trauma or distress [14–17, 32] and (iv) fears that it could result in reductions in staffing and one-to-one contact between staff and patients on wards.

In order to plan effective and safe mental health service delivery, it is important to determine whether evidence supports the use of surveillance technologies. Both potential benefits and harms should be considered, including impacts on outcomes such as safety, care quality, mental health and treatment satisfaction. However, a comprehensive review of the evidence underpinning the use of surveillance technologies in inpatient settings has not yet been undertaken. Therefore, we conducted, to our knowledge, the first systematic review of a range of surveillance technologies in inpatient mental health settings.

Both quantitative and qualitative evidence is synthesised to answer the following overarching research question: how are surveillance-based technology initiatives being used and implemented in inpatient mental healthcare settings, and what is their impact? Our specific three research objectives were as follows:

1. How are surveillance-based technologies in inpatient mental health settings being implemented and what are the related implementation outcomes?
2. How are surveillance-based technologies in inpatient mental health settings experienced (e.g. by patients, staff, carers, visitors)?
3. What is the effect, including benefits, harms and unintended consequences, of surveillance-based technologies in inpatient mental health settings for outcomes such as patient and staff safety and patient clinical improvement?

## Methods

We conducted a systematic review following the Preferred Reporting Items for Systematic Reviews and Meta-Analyses (PRISMA) statement [33]. The PRISMA checklist can be found in Appendix A [see Additional File 1]. The protocol for our review was registered with PROSPERO (CRD42023463993). Amendments to this protocol are described in Appendix B, with justifications [see Additional File 1].

This review was conducted by the National Institute for Health and Care Research (NIHR) Policy Research Unit in Mental Health (MHPRU) based at King’s College London and University College London, which conducts research in response to policymaker need (e.g. in the Department of Health and Social Care or NHS England). Our working group met weekly, and included academic and lived experience researchers, and clinicians.

### Lived experience involvement

The working group included five lived experience researchers, who took part in all stages of the research from design, screening and extraction to analysis and write-up. The lived experience researchers included people with experience of inpatient care; conducting patient-led ward inspections; peer advocacy and support; being a carer; and direct experience of surveillance technologies during admission to inpatient mental health services. Some of the lived experience researchers were in liaison with service user groups and patients with experience of surveillance technologies. Due to the sensitive nature of the topic and related experiences, some lived experience researchers in the group have chosen to remain anonymous. Two experts by experience who had direct experience of surveillance technologies, including in an inpatient mental health setting, contributed to the lived experience commentary.

### Search strategy

We searched five electronic databases (Embase, MEDLINE, PsycInfo, PubMed and Scopus) for peer-reviewed literature relevant to our research objectives. We searched for grey literature relevant to research objective 2 on a grey literature database (the Health Management Information Consortium) and two pre-print servers (medRxiv and PsyArXiv). Database searches were initially conducted between 17/09/2023 and 18/09/2023, with no date or language restrictions and were updated on 19/09/2024. Screening of non-English language papers was conducted using Google Translate; extraction and quality appraisal of full texts was conducted by someone with knowledge of the language. We contacted experts (including from NHS England, the Care Quality Commission and research experts internationally) to request additional literature we may not have identified. Our lived experience networks supported the identification of additional grey literature. We also reference list screened and citation tracked included studies and relevant systematic reviews. Our search strategy included key terms relating to surveillance and inpatient mental health settings, as detailed in Appendix C [see Additional File 1].

### Screening

Title and abstract and full text screening were conducted in Rayyan [34]. Title and abstract screening was conducted by seven researchers (KS, UF, JG, AG, CR and two NIHR MHPRU Lived Experience Researchers). One hundred percent of titles and abstracts were independently double screened. Full text screening was conducted by nine researchers (KS, UF, JG, AG, CR, RC and three NIHR MHPRU Lived Experience Researchers). One hundred percent of full texts were independently double screened. Any disagreements were resolved by discussion between KS, UF, JG and AG.

### Eligibility criteria

#### Participants

Participants include mental health patients (of any age, sex or gender), staff, carers and visitors to inpatient mental health services, Section 136 suites and places of safety.

#### Intervention

Surveillance-based technology initiatives include CCTV, remote monitoring initiatives, smart watches and body-worn cameras. We excluded studies which focused solely on door locking, door security, or key card access practices and policies, without explicit reference to surveillance technologies.

#### Comparators/controls

Studies with or without any type of comparator or control group were eligible for inclusion.

#### Outcomes

For research objective 1 we included studies which mapped where, when, how, how often and by whom such surveillance initiatives are used and who they are used on. Information related to lived experience involvement in the development, implementation, use and evaluation of the intervention was also included, as were implementation outcomes including appropriateness, adoption, feasibility, fidelity, sustainability, penetration and costs.

For research objective 2, we included studies which reported qualitative data on patient, staff and family/carer pre-implementation perceptions and post-implementation experiences of surveillance technologies.

For research objective 3, we included quantitative data on outcomes including safety of patients, staff, carers and visitors, use of restrictive practices and other containment measures, cost-effectiveness, care quality outcomes, clinical mental health outcomes, wellbeing and satisfaction of patients, staff, carers and visitors.

#### Setting

Inpatient mental health/psychiatric hospitals, Section 136 suites and places of safety were included. Inpatient mental health services admit patients, either voluntarily or involuntarily, for at least one night to receive treatment for mental health difficulties. Inpatient mental health services with any length of stay were eligible for inclusion, ranging from acute inpatient mental health wards offering shorter-term support to longer-term rehabilitation wards and forensic wards. We included general inpatient mental health wards, as well as those for any specific psychiatric diagnoses. A “place of safety” is a designated location, such as a hospital or police station, where individuals detained under mental health legislation are taken by police for mental health assessment. Section 136 suites are a specific type of place of safety, typically a dedicated area within a hospital. We excluded studies based in emergency departments, dementia-specific wards, care/nursing homes, outpatient and drop-in crisis services.

#### Design

We included all study designs reporting quantitative, qualitative and mixed methods data. We excluded conference proceedings, abstracts without an associated full text, books, PhD/MSc/BSc theses, opinion pieces, reviews, blog posts and social media content. For grey literature to be eligible for inclusion in relation to research objective 2, the sources had to, at least briefly, describe their methodological approach. No language or location restrictions were imposed during our searches or screening.

### Data extraction

A data extraction sheet was designed in Microsoft Excel and revised based on feedback from the working group and piloting on an eligible paper by JG. The final data extraction sheet template can be seen in Additional File 2. Data extraction was conducted by eight researchers (KS, JG, UF, AG, CR, RC and two NIHR MHPRU Lived Experience Researchers). Data were independently double extracted for 4/32 (12.5%) of the included papers and an expert quantitative researcher (ARG) checked the accuracy and interpretation of all quantitative data extracted.

### Quality appraisal

As the included studies varied in design, we used the Mixed Methods Appraisal Tool (MMAT) to assess quality [35]. We also noted any additional ethical issues, the degree of lived experience involvement in the studies, and conflicts of interest reported in the papers, such as author affiliations with surveillance technology companies or funding received from them. Potential undisclosed conflicts of interest were also investigated through online searches of authors using search engines. Quality appraisal was conducted by nine researchers (KS, JG, UF, AG, CR, RC, PB and two NIHR MHPRU Lived Experience Researchers). Independent double quality appraisal was conducted for 9/32 (28.1%) of the included papers. Any discrepancies in quality appraisal ratings were resolved by discussion and involvement of a third review author where necessary.

### Evidence synthesis

Evidence synthesis was led by JG and UF. The interpretation of data and synthesis of results was supported by KS and the working group. Data were synthesised by research objective, and study characteristics were tabulated. Where possible, results were reported separately by type of surveillance technology.

Synthesis methods by research objective:

1. *Implementation mapping and outcomes*: We mapped the way the surveillance-based technologies were used in our settings of interest by technology type, including details (where available) on where, when, how often and by whom surveillance-based technologies are used and who was being surveilled. We tabulated and narratively described [36] implementation outcomes including appropriateness, adoption, feasibility, fidelity, costs, penetration and sustainability [37].
2. *Perceptions and experiences*: Quantitative and qualitative data documenting perceptions and experiences of surveillance technologies were narratively synthesised [36]. We synthesised data separately according to whether perceptions and experiences were reported pre- or post-implementation of surveillance technologies. Findings were grouped into benefits and potential uses, concerns and potential harms, or neutral views, and then by respondent (e.g. patients, staff, family/carers) where possible.
3. *Quantitative measures of effect*: Quantitative outcome data were tabulated and summarised narratively [37]. This included reporting original measures of effect (e.g. risk ratios, odds ratios, or risk differences for dichotomous outcomes, and mean differences or standardised mean differences for continuous outcomes) and *p*-values, where available. Results were grouped according to surveillance technology type. We were unable to perform a meta-analysis due to heterogeneity across the types of outcomes, measures of effect, populations and length of follow-up.

## Results

**Fig. 1:**
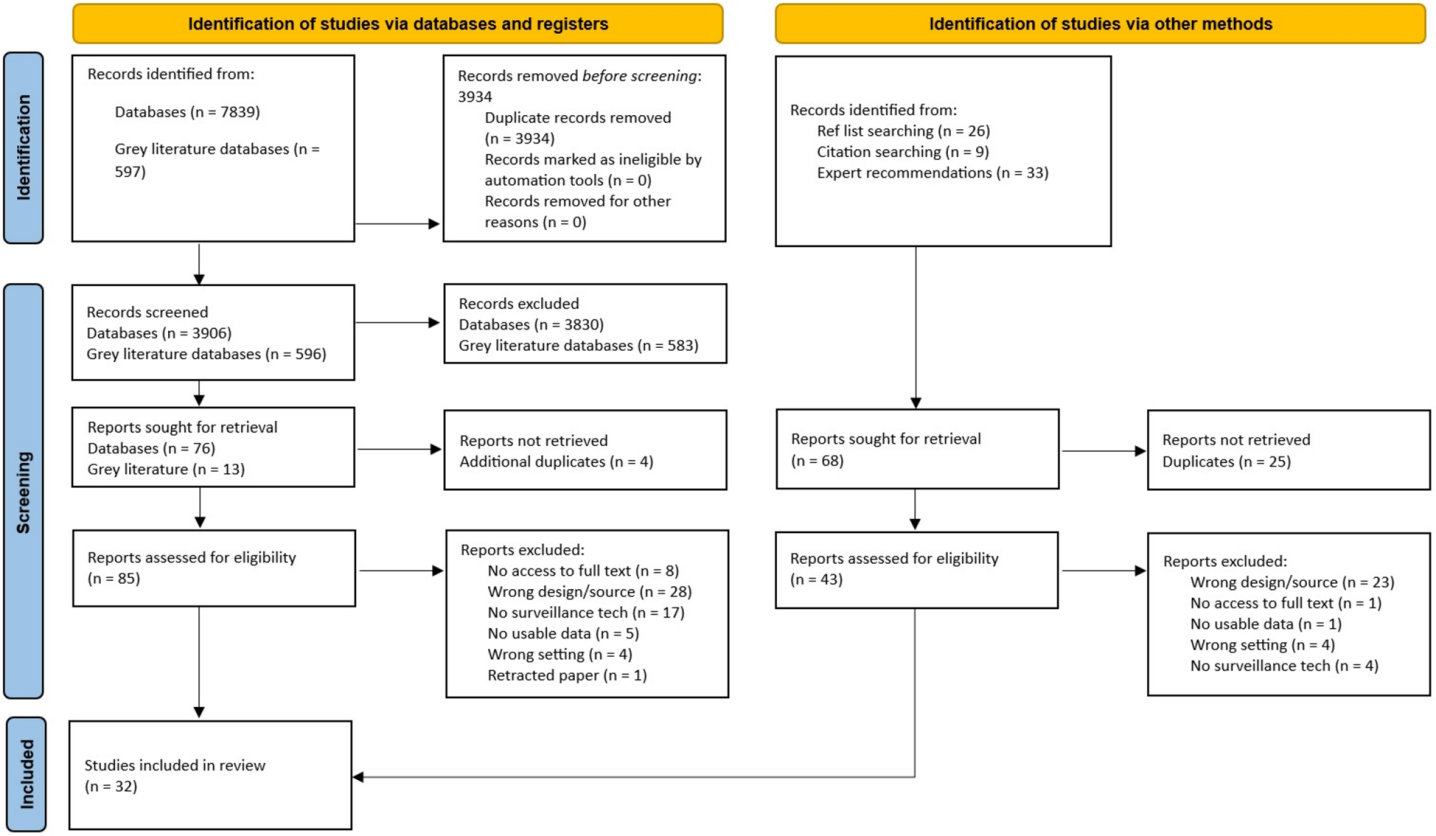
PRISMA flow diagram

Figure 1 presents the PRISMA flow diagram [33] of the screening and selection process. We identified 32 studies for inclusion. Excluded full texts and their reasons for exclusion can be seen in Appendix D [see Additional File 1]. Approximately two-fifths of included studies reported on CCTV/video monitoring (*n* = 13), other studies reported on VBPMM (*n* = 9), BWCs (*n* = 6), GPS electronic monitoring (*n* = 2) or wearable sensors (*n* = 2). Most studies were conducted in the UK (*n* = 23), with two conducted in Germany, one multi-country study and one each conducted in Ireland, Malaysia, Finland, Australia, Israel and USA. Fifteen (46.9%) studies were quantitative in design, eight (25.0%) qualitative and nine (28.1%) mixed methods. Most studies reported data from a mix of ward types (*n* = 11), followed by acute wards (*n* = 7), low/medium secure wards (*n* = 5), forensic wards (*n* = 5) and psychiatric intensive care units (PICUs) (*n* = 4). Only two studies specified that they included wards with inpatients under the age of 18 [38, 39]. The remaining studies either exclusively focused on inpatient wards for adults or did not specify the age of the inpatient populations.

Sixteen papers (50.0%) were rated as low quality, five studies (15.6%) were rated as medium quality and eleven studies (34.4%) were rated as high quality. For full details on MMAT ratings, see Additional File 3. Nine papers (28.1%) disclosed conflicts of interest. One report was produced by a surveillance technology company [39], whilst other conflicts of interest included the project being funded by a surveillance technology company [40–43], authors’ time or expenses being funded by a technology company [44, 45], technology companies contributing to the conduct of the study [40–43, 46] or authors working for a surveillance technology company [40, 45–47]. In one of these papers, authors were listed as being affiliated with a technology company, but this was not reported as a conflict of interest in the declarations section [47]. Out of the 32 studies included in this review, we also identified a potential undeclared conflict of interest in one study, involving an author receiving prior funding from organisations involved in the use of surveillance technologies. Study characteristics, including quality ratings, are summarised in Table 1. A more detailed version of this table is provided in Appendix E [see Additional File 1].

**Table 1:** Table of study characteristics

Author, year and country	Study aims	Surveillance description	Study design	Inpatient setting	Sample (including control group)	Lived experience involvement	MMAT quality rating	Conflicts of interest
Barrera et al. 2020 [47] Country: England	Establish whether it is safe to conduct nursing observations remotely from the nursing office using VBPMM.	VBPMM; Oxevision by Oxehealth	Service improvement project/feasibility study Pre-post design with a concurrent control period in the initial implementation phase, where VBPMM-assisted observations were compared to treatment as usual.	An adult acute male inpatient mental health ward.	Patients, staff and relatives Patients *n* = not reported Staff *n* = 18 Relatives *n* = 10 Total *n* = unknown	Yes	Low	Yes
Bowers et al. 2002 [48] Country: England	Describe current safety and security measures used on acute psychiatric wards in London, and to explore the relationships between them.	CCTV/video surveillance; CCTV for security (location on ward not specified); brand(s) not specified	Quantitative survey	Acute psychiatric wards in London. Age of the inpatient population not specified.	*N* = 87 hospital wards	No	Low	No
Buckley et al. (2024) [41] Country: England	Adapt a previous health economic model with clinical data collected from additional NHS mental health trusts, and to present the results in an accessible format to inform healthcare decision makers.	VBPMM; Oxevision by Oxehealth	Economic analysis study utilising a cost calculator approach (using observational data collected across five English NHS mental health trusts)	Acute adult inpatient mental health and older adult inpatient mental health services across five mental health NHS trusts which wished to remain anonymous.	Patients and staff *n* = not reported	No	Low	Yes
Clark et al. (2021) [40] Country: England	Primary aim: Improve the quality of physical health monitoring by making accurate vital sign measurements more frequently available. Secondary aim: Explore the clinical experience of integrating a technological innovation with routine clinical care.	VBPMM; Oxevision by Oxehealth	Proof of concept quality improvement project	A women’s PICU in a hospital in South London. Age of the inpatient population not specified.	Staff, patients and carers Patients in pre-implementation focus group *n* = 12 Patients surveyed post-surveillance in seclusion *n* = 12 Carers surveyed post surveillance in seclusion *n* = 6 Staff *n* = not reported; Total *n* = unknown	Yes	Low	Yes
Curtis et al. (2013) [49] Country: England	Evaluate a purpose built inpatient mental health care facility, the “New Hospital”.	CCTV/video surveillance; CCTV cameras in common areas; brand(s) not specified	Qualitative evaluation	The “New Hospital” had 318 inpatient beds to care for patients with acute psychiatric illnesses, geriatric conditions, learning difficulties, and a significant number of forensic cases. Age of the inpatient population not specified.	Staff, patients, family and carers Results are reported from 19 group or individual meetings, representing a subset from a total of 40 conversations in the wider study. It is unclear why this subset was selected Number of participants = not reported	No	High	No
Dewa et al. 2023 [32] Country: England	Conduct a qualitative service evaluation to explore both staff and patient perspectives on the use of Oxehealth technology in a high-secure forensic psychiatric hospital.	VBPMM; Oxevision by Oxehealth	Qualitative study	Broadmoor Hospital in South England within West London NHS Trust—an adult high-secure forensic inpatient service.	Staff and patients Patients *n* = 12 Staff *n* = 12 Total *n* = 24	Yes	High	No
Due et al. 2012 [50] Country: Australia	Explore the potential relationship between surveillance techniques, the enactment of security measures, and patient violence in mental health wards.	CCTV/video surveillance; CCTV or surveillance cameras in all areas on each ward except bedrooms and bathrooms; brand(s) not specified	Ethnographic case study	The mental health unit of a large public hospital in South Australia. The buildings comprised both a secure or “locked” ward, and an open ward. Age of the inpatient population not specified.	Patients, staff, visitors	No	High	No
Ellis et al. 2019 [44] Country: England	Conduct a pilot project to evaluate whether issuing BWCs to mental health ward nurses was associated with a reduction in violence and aggression in recorded incident interventions.	BWCs; brand was Reveal trading as Calla	A quasi-experimental repeated measures design	Seven West London Trust mental health adult wards, including: two wards for local services admissions (male and female), a PICU (male), a low secure forensic ward (male), medium secure ward (female) and two enhanced medium secure wards (both female).	Staff and patients Staff who completed the pre-pilot questionnaire *n* = 63 Patient *n* = not reported Total *n* = unknown	No	Low	Yes
Foye et al. (2024a) [51] Country: England	Explore the perspectives of patients, mental health staff, and senior management to understand the practical and ethical issues related to the implementation of BWCs.	BWCs; brand was Reveal trading as Calla	Qualitative study	Five NHS acute adult inpatient wards across England.	Staff and patients Total *n* = 64 Patients via NHS services n = 24 Staff via NHS services *n* = 25 Service users via social media *n* = 9 Mental health nursing directors *n* = 6	Yes	High	No
Foye et al. (2024b) [52] Country: England	Evaluate the implementation of BWCs on two inpatient mental health wards, including the impact on incidents, the acceptability to staff and patients, the sustainability of the resource use and ability to manage the use of BWCs on these wards.	BWCs; brand was Reveal trading as Calla	Mixed methods study with a repeated measures design	One adult acute ward and one adult psychiatric intensive care unit in a London mental health trust.	Staff and patients Total *n* = 22 Interviewed patients *n* = 5 Interviewed staff members *n* = 17	Yes	Low	No
Greer et al. 2019 [53] Country: England	Explore the attitudes of staff towards passive remote monitoring technology for risk of aggression in inpatient forensic mental health services, with a focus on the potential benefits that this technology could provide and barriers to implementation.	Wearable sensors; brands were E4 (Empatica Srl) and Everion (Biovotion Ltd)	Qualitative study using focus groups	Medium-secure forensic mental health service in South London, UK. Age of the inpatient population not specified.	Staff (*n* = 25)	Yes	High	No
Hakimzada et al. 2020 [54] Country: England	Explore the attitudes of psychiatric nursing staff towards the use of BWCs on psychiatric inpatient wards.	BWCs; brand(s) not specified	Quantitative and qualitative survey questionnaire	Seven inpatient wards in one Mental Health Trust in South West London, including a PICU, two acute wards and four secure wards. Age of the inpatient population not specified.	Staff (*n* = 60)	No	Medium	No
Hardy et al. 2017 [55] Country: England	Examine the feasibility of using BWCs in an inpatient mental health setting.	BWCs; brand was Reveal trading as Calla	Mixed methods pre-post pilot study	Berrywood Hospital, an adult psychiatric facility in Northampton, England, run by Northamptonshire Healthcare NHS Foundation Trust. The five wards in the pilot included one male and one female recovery, one low secure unit, one acute.	Patients and staff Number of participants = not reported	No	Low	No
Krieger et al. 2018 [38] Country: Germany	Assess patients’ preferences regarding prevalent specific forms of coercive interventions, their accompanying emotions, and their understanding of the experience as measured at different sites and different points in time using both interviews and self-assessments.	CCTV/video surveillance; part of the questionnaire specifically asks about patients’ preferences for video surveillance in seclusion; brand(s) not specified	Naturalistic trial	Three PICUs at the Asklepios Clinic North in Hamburg, Germany. Age of the inpatient population not specified. However, it can be inferred that patient participants included adults and children.	Patients Patients in coercive intervention group *n* = 213 Patients in comparison group (voluntary admission with no coercive treatment) *n* = 51	No	Medium	No
Malcolm et al. 2022 [42] Country: England	To explore the impact of introducing VBPMM with standard care, versus standard care alone on health and economic outcomes in PICUs across England.	VBPMM; Oxevision by Oxehealth	Economic analysis study utilising a cost-calculator approach (using data from a single centre observational before and after study)	An adult PICU	Patients (*n* = not reported)	No	Low	Yes
Malcolm et al. 2022 [43] Country: England	To assess the costs and outcomes associated with introducing VBPMM in an acute adult mental health inpatient hospital and an older adult mental health inpatient hospital for dementia.	VBPMM; Oxevision by Oxehealth	Economic analysis study utilising a cost calculator approach (using observational data collected at two English NHS mental health trusts)	An acute adult mental health inpatient hospital and an older adult mental health inpatient hospital for dementia at Coventry and Warwickshire Partnership NHS Trust in England	Patients (*n* = not measured) Interviews conducted with four staff (a ward manager, ward matron, a nurse, and a consultant psychiatrist) to inform figures for staff time needed to manage self-harm events on acute adult wards, and nighttime bedroom falls in older adult wards.	No	Low	Yes
Murphy et al. 2017 [56] Country: England	To compare the costs of using GPS electronic monitoring (EM) in forensic psychiatric patients on leave from a medium-secure service by comparing the average total cost per patient with electronic monitoring against the average total cost per patient without EM.	GPS electronic monitoring; brand(s) unspecified	Retrospective observational study	River House, an adult medium-secure unit in South London and Maudsley NHS Foundation Trust (107 male beds and 15 female beds)	Patients Intervention group *n* = 121 Control group *n* = 96 Total patients *n* = 175 Comparison group was patients who had used leave during a 3-month period in 2010 (no electronic monitoring). Intervention group was patients who had used leave in the corresponding period in 2011 (during which electronic monitoring had been implemented).	No	Medium	No
Ndebele et al. 2022 [46] Country: England	To assess the effect of clinical teams augmenting their existing clinical practices with a VBPMM system on the number of assaults and rapid tranquillisation events.	VBPMM; Oxevision by Oxehealth	Single-centre pragmatic study	A male PICU ward at Coventry and Warwickshire NHS Partnership Trust. Age of patient population not stated.	Staff interviews: *n* = 4 Staff surveys: *n* = 8	No	Low	Yes
Ndebele et al. 2023 [45] Country: England	To examine the effect of adopting the contact-free VBPMM system into existing clinical practice on the number of incidents of self-harm in bedrooms (all types and ligatures specifically) on acute mental health inpatient wards. A minor aspect of the study was to include patient and staff feedback.	VBPMM; Oxevision by Oxehealth	Mixed methods non-randomised controlled before-and-after evaluation within a pilot study	At Caludon Centre, Coventry & Warwickshire Partnership NHS Trust (CWPT), a purpose-built facility, based on the University Hospital Coventry and Warwickshire (UHCW) site, providing inpatient and outpatient adult mental health care.	Staff and patients Number of patients in total = not reported Intervention group: two acute wards fitted with VBPMM (22-bed female and 20-bed male) Control wards: two acute wards without VBPMM selected based on the similarity of the patient cohort, ward size and clinical ways of working	No	Low	Yes
Nijman et al. 2011 [57] Country: England	To investigate the prevalence of door locking and the use of other exit security measures on psychiatric admission wards in the UK, and to empirically study the associations between locking ward exit doors and absconding rates.	CCTV/video surveillance; CCTV; brand(s) not specified	Cross sectional study	133 adult acute psychiatric wards in London, Central England and Northern England which participated in the City-128 study [58].	Staff responded to the survey. Individual wards were the unit of measurement	No	High	No
Oxehealth, 2022 [39] Country: England	Not clearly stated	VBPMM; Oxevision by Oxehealth	Mixed methods study	13 wards, including the following services: female working age acute, male working age acute, mixed working age acute and psychiatric intensive care units (age not specified).	Patients (*n* = “over 75”) Number of patients rating each statement ranged from 60 to 78. “No opinion” responses were not included in these counts. Specific overall number of participants not stated	No *However, in this report there is a description of the wider PPI work undertaken by Oxevision*	Low	Yes
Peek-Asa et al. 2009 [59] Country: USA	Compare the workplace violence prevention programmes in a sample of psychiatric units and facilities in New Jersey and California. The units and facilities were compared on four components: training, policies and procedures, environmental safeguards, and security.	CCTV/video surveillance; CCTV brand(s) not specified	Cross sectional survey	83 psychiatric units within acute care hospitals and psychiatric facilities in New Jersey and California. Age of the inpatient populations not specified.	Psychiatric units were the individual unit of analysis. 53 in California 30 in New Jersey	No	Low	No
Shetty et al. 2023 [60] Country: Ireland	Explore the patients’ experiences of different observation methods in seclusion and their influence on their connection and relations to staff, by patients in an Irish forensic mental health hospital, in order to inform future seclusion practices.	CCTV/video surveillance; video camera in seclusion room; brand(s) not specified	Retrospective phenomenological qualitative study	Medium secure wards (three male, one female) at an adult forensic mental health hospital in Ireland.	Patients (*n* = 10)	No	High	No
Simpson et al. 2011 [61] Country: England	Discover whether rates of drug/alcohol use on acute psychiatric wards were related to levels and intensity of exit security measures.	CCTV/video surveillance; CCTV brand(s) not specified	Cross-sectional study	136 acute adult psychiatric wards across London, Central England and North England.	Same as Nijman et al. [57]	No	High	No
Steinert et al. 2014 [62] Country: Germany	Conduct an online survey on the current practice of coercive measures in German psychiatric hospitals, in light of regional legal prohibition of video surveillance (Nordrhein-Westfalia) in 2011.	CCTV/video surveillance; video monitoring during physical restraint; brand(s) not specified	Cross-sectional survey (online questionnaire)	88 psychiatric hospitals in Germany This includes 36 specialist hospitals, 41 departments within general hospitals and 13 university hospitals. These included general psychiatry hospitals, as well as those for addictions, forensic psychiatry and old-age psychiatry. Age of the inpatient populations not specified.	Staff (*n* = 88)	Yes	High	No
Tapp et al. 2016 [63] Country: Multi-country	Establish whether experts with clinical and/or research experience in this setting could reach consensus on elements of high-security hospital services that would be essential to the rehabilitation of forensic patients.	CCTV/video surveillance; CCTV brand(s) not specified	Three-round Delphi study	Forensic high security inpatient mental health services. Age of the inpatient population not specified.	Staff (*n* = 54)	No	Medium	No
Tron et al. 2018 [64] Country: Israel	i) Develop and evaluate a framework for using wearable devices to facilitate continuous motor deficits monitoring in schizophrenia patients in a natural setting. ii) Help characterise subtypes of schizophrenia to better understand its causes and develop more personalised treatments.	Wearable sensor; smartwatch (GeneActiv) worn by patients with psychosis	Quantitative evaluation	Closed adult inpatient wards at Shaar-Meashe mental health centre.	Patients (*n* = 25)	No	Low	No
Tully et al. 2016 [65] Country: England	Determine whether the introduction of Electronic Monitoring (EM) using GPS “tracking” led to a reduction in episodes of leave violation. They also aimed to assess the extent to which electronic monitoring affected the amount of overall leave and the proportion of leave that was unescorted.	GPS electronic monitoring; brand was “Buddi Tracker”	Observational pre-post study	The South London and Maudsley medium secure service in England (comprising two medium secure units in South London at the time of the study). Age of the inpatient population not specified.	N/A	No	Low	No
Vartianinen & Hakola, 1994 [66] Country: Finland	To study, with a questionnaire, the effects of TV monitoring on patients and personnel.	CCTV/video surveillance; brand(s) not specified	Pre-post study using a survey	Four closed adult male wards in the Niuvanniemi hospital in Finland.	Staff and patients Staff *n* = 97 Patients *n* = 77	No	Low	No
Warr et al. 2005 [67] Country: England	Determine the acceptable use of CCTV surveillance in a mental health inpatient unit and whether it benefits patient care.	CCTV/video surveillance in bedrooms; brand(s) not specified	Qualitative interview study	Montpellier adult low-secure unit in England.	Staff and patients Staff *n* = 10 Patients *n* = 6	No	Medium	No
Wilson et al. 2023 [68] Country: England	Explore the perspectives of patients, mental health staff, and senior management to identify the possible impacts of BWCs in inpatient mental health settings.	BWCs; brand(s) not specified	Explorative qualitative study	Five NHS acute adult inpatient wards across England.	Staff and patients Total *n* = 64 Staff *n* = 25 Patients *n* = 24 Service users from Twitter *n* = 9 Mental health nursing directors *n* = 6	Yes	High	No
Zakaria & Ramli, 2018 [69] Country: Malaysia	Identify patients’ perceptions of physical privacy dimensions proposed by Carew and Stapleton.	CCTV/video surveillance; brand(s) not specified	Qualitative study	Psychiatric wards at a teaching hospital in Malaysia (included child and adult inpatients).	Patients (*n* = 25)	No	High	No

Acronyms: *BWCs* body-worn cameras, *CCTV* closed circuit television, *EM* electronic monitoring, *GPS* Global Positioning System, *MMAT* Mixed Methods Appraisal Tool, *NHS* National Health Service, *PICU* psychiatric intensive care unit; *UK* United Kingdom, *USA* United States of America, *VBPMM* Vision-Based Patient Monitoring and Management

### Research objective 1: how are surveillance-based technologies in inpatient mental health settings being implemented and what are the related implementation outcomes?

Below we have summarised how surveillance technologies have been implemented, and reported implementation outcomes, by type of surveillance technology. Full details on implementation process, setting, informed consent procedures and lived experience involvement can be found in Appendix F [see Additional File 1] whilst implementation outcomes can be found in Table 2.

**Table 2:** Summary of implementation outcomes (appropriateness, feasibility, fidelity, adoption, sustainability, penetration, costs) across the surveillance technologies

Surveillance technology	Implementation outcome	Results	MMAT quality rating	Conflicts of interest
Vision Based Patient Monitoring and Management (VBPMM)	Feasibility (*n* = 1 paper)	• **Ndebele et al.** [45]**:** Oxevision consent rates were 67% for the female acute ward, and 76% for the male acute ward.	Low	Yes
	Fidelity (*n* = 1 paper)	**Barrera et al.** [47]**:** • There were no significant gaps or drops in the use of Oxevision during the 4-week evaluation. • On a few nights, usage was slightly lower than expected, so some staff became “sensor champions” to ensure all staff on each night shift were trained to use it. • During the first four night shifts, staff performed and recorded their observations as required.	Low	Yes
	Penetration (*n* = 1 paper)	**Barrera et al.** [47]**:** • 17,299 observations over an estimated 755 patient nights had been monitored. After 4 months, 41 patients had spent on average 14.58 (SD 14.55) nights in bedrooms with sensors (minimum of one night and maximum of 86 nights).	Low	Yes
	Costs (*n* = 3 papers)	• **Malcolmet al.** [42] provided the following breakdown of costs of VBPMM in a psychiatric intensive care unit (PICU) setting: Clinical inputs and cost estimates were informed by data from an unpublished observational study on Oxevision conducted on a PICU ward, data provided by Oxehealth and targeted searching to inform different parameters, and publicly available sources such as NHS reference costs and Personal Social Services Research Unit. Further details are provided in the paper. **Scaling inputs used to scale populations (clinical study specific input, average sized ward input, NHS input)** informed by Hospital Episodes Statistics, observational study data, data from Oxehealth, and NHS data: Average length of stay per patient (days): 48, 48, 48 Number of wards: 1, 1, N/A Average beds per ward: 11, 12, 12 Average occupancy: 91%, 90%, 90% Total occupied bed days per year: 3648, 3945, 302,427 **Clinical inputs used in the model:** Night-time observation hours: Number of night-time observations per occupied bed day: 28.8 Night-time observation time per patient (seconds): 23 Reduction in observation time from VBPMM: 41% (95% confidence interval: 25–53%) One-to-one observation hours: Number of one-to-one observation hours per occupied bed day: 7.39 Reduction in one-to-one observation hours from VBPMM: 7% (− 41%) Proportion of one-to-one observation hours that is cash releasing: 78% Assaults: Average number of assaults per occupied bed day: 0.0125 Reduction in assaults from VBPMM: 26% (− 18%) Staff hours per incident: 7.3 Proportion visiting A&E: 2% Rapid tranquillisation events: Average number of rapid tranquillisation events per occupied bed day: 0.0253 Reduction in rapid tranquillisation events from VBPMM: 40% (10%) Staff hours per incident: 10.1 Complication risk from using tranquillisers: 3% **Cost inputs:** Night-time observation hours: Weighted staff cost for observation time per hour: £30 One-to-one observation hours: Weighted staff cost for observation time per hour: £30 Assaults: Weighted staff cost per hour: £45 Cost of A&E (non-surgery): £2,134 Rapid tranquillisation event: Weighted staff cost per hour: £43 Cost of tranquillisers: £5 Cost of tranquilliser complication: £716 VBPMM Cost of VBPMM (average sized ward): £26,190 (taking into account annual licence fees, installation cost and cabling costs provided by Oxehealth, and staff training costs calculated by combining staff costs from the Personal Social Services Research Unit with estimated staff numbers requiring training per ward provided by Oxehealth) **Outcomes generated by the model (Standard care, Oxevision + standard care, Difference):** Cost of night-time observational hours: £268, £158, − £109 Cost of one-to-one observation hours: £10,749, £9,943, − £806 Cost of assaults: £227, £167, − £60 Cost of rapid tranquillisation event: £562, £338, − £223 Cost of VBPMM £0, £319, £319 Total cost per patient £11,806, £10,926, − £880 Total cost per occupied bed day £246, £228, − £18 Total cost per average sized ward per year £970,193, £897,907, − £72,286 Total cost to the NHS per year £74,381,491, £68,839,567, − £5,541,924 • **Malcolm et al.** [43] provided the following breakdown of their cost-calculator analysis of VBPMM on two adult acute inpatient mental health wards: Model inputs were informed by two observational studies on the clinical effectiveness of VBPMM at Coventry and Warwickshire Partnership NHS Trust (one published, one unpublished) and by literature searches to inform specific parameters. Costing was based on standard UK sources from the NHS Reference Costs, Personal Social Services Research Unit and figures from Oxevision. **Scaling inputs for application of model results (study-specific input, average ward size input, NHS input)** based on Hospital Episode Statistics, observational studies, data from the Trust’s commissioning support unit, and some assumptions): Average length of stay per patient (days): 48, 48, 48 Number of wards: 2, 1, N/A Average beds per ward: 21, 16, 16 Average occupancy: 95%, 90%, 90% Total occupied bed days per year: 14,572, 6903, 3,931,551 **Clinical inputs into the model** (obtained from the two observational VBPMM studies and interviews with four mental health hospital staff members): Night-time observation hours: Number of night-time observations per occupied bed day: 11.52 Night-time observation time per patient (seconds): 23 Reduction in observation time from VBPMM: 41% (95% confidence interval: 25% to 53%) One-to-one observation hours: Number of one-to-one observation hours per occupied bed day per year: 1.25 Reduction in one-to-one observation hours from VBPMM: 20% (95% confidence interval: -13% to 36%) Proportion of one-to-one observation hours that is cash releasing: 40% Self-harm incidents: Average number of self-harm incidents per occupied bed day per year: 0.0094 Reduction in self-harm incidents from VBPMM: 44% (95% confidence interval: 13% to 100%) Staff hours per incident: 9.3 Proportion visiting A&E: 8% **Cost inputs into the model were** (based on standard UK sources from the NHS Reference Costs and Personal Social Services Research Unit, and figures from Oxevision) Night-time observation hours: Weighted staff cost for observation time per hour: £30 One-to-one observation hours: Weighted staff cost for observation time per hour: £30 Self-harm incidents (adults on acute mental health wards only): Weighted staff cost per hour: £37 Cost of A&E (non-surgery): £2134 Tariff cost of A&E (non-surgery): £1604 Cost of VBPMM on an average sized adult acute mental health ward: £26.542 (taking into account annual licence fees and annuitised installation and cabling costs provided by Oxevision and staff training costs). **Clinical events per patient per year (standard care, VBPMM + standard care, incremental):** Total staff hours per patient: 68.16, 52.68, − 15.48 Assaults or self-harm incidents per year: 0.53, 0.34, − 0.20 A&E visits per patient: 0.04, 0.02, − 0.02 **Best case results:** VBPMM (standard care cost, VBPMM + standard care cost, incremental): Cost of observations (£ per patient): £107, £63, − £44 Cost of one-to-one observations (£ per patient): £1813, £1444, − £369 Cost of self-harm incidents (£ per patient): £231, £129, − £101 Cost of VBPMM (£ per patient): £0, £242, £242 Total cost per patient (£): £2151, £1879, − £272 Total cost per occupied bed day (£): £45, £39, − £6 Total cost per average sized ward per year (£): £235,676, £205,849, − £29,827 Total cost to the NHS per year (£): £176,167,573, £153,872,139, − £22,295,434 • **Buckley et al.** [41] provided the following breakdown of their cost-calculator analysis of VBPMM, using data from five before-and-after studies at five mental health NHS Trusts, including acute adult mental health inpatient wards and older adult mental health inpatient wards. The data used was scaled to an average ward (assumed to be 16 beds at 90% occupancy) Cost inputs were extracted from publicly available sources such as Personal Social Services Research Unit (SSRU) and NHS Cost Collection. **Summary of acute mental health services** Trust 1: 1 × 16 bed ward Trust 2 (Coventry and Warwickshire Partnership NHS Trust): 2 wards, 42 beds in total Trust 3: 1 × 18 bed ward Trust 4: 1 × 18 bed ward, 1 × 16 bed wards, 1 × 17 bed ward **Summary of older adult mental health services** Trust 1: 1 × 12 bed ward, 1 × 16 bed ward Trust 2 (Coventry and Warwickshire Partnership NHS Trust): 2 × 24 bed wards Trust 5: 1 × 24 bed ward, 1 × 14 bed ward, 1 × 17 bed ward **Cost inputs:** Night time observation hours (from PSSRU and NHS Cost Collection 2021/22): Weighted staff cost for observation time per hour: £34 One-to-one observation hours (from PSSRU and NHS Cost Collection 2021/22): Weighted staff cost for observation time per hour: £34 Self-harm incidents (adults on acute mental health ward only) (from PSSRU and NHS Cost Collection 2021/22): Weighted staff cost per hour: £42 Cost of A&E (non-surgery): £2380 (based on weighted average of fracture codes (HE11E-HE71D) without interventions, using NHS Reference costs 2021/22) Bedroom falls at night (older adult population only) (from PSSRU, NHS Cost Collection 2021/22 and a 2017 NHS Improvement report): Weighted staff cost per hour: £70 Increased length of stay cost from the resulting fall: £3458 Cost of GP review: £123 Litigation cost: £15,183 Staff cost per hour for A&E visits: £28 A&E cost (non-surgery treatment): £2380 (using weighted average of fractures codes HE71B-HE11H without interventions, based on NHS Reference Costs 2021/22). Hospital stay without surgery cost: £341 Hospital stay with surgery cost: £2726 Surgery cost: £4869 Cost of emergency service callout: £348 VBPMM: Cost of VBPMM for an average-sized adult acute mental health inpatient ward: £29,457 Cost of VBPMM for an average-sized older adult mental health inpatient ward: £29,472 These VBPMM cost estimates include annual licence fees and annuitized installation and cabling costs provided by Oxevision, and including staff training costs calculated by combining staff costs from PSSRU with estimated training requirements provided by Oxevision—this input is higher in the older adult population due to more staff requiring the training. **Clinical inputs:** **NHS trust data used to populate the model:** Night-time observation time: Older adult mental health services: 1 NHS trust (1 ward) Adult acute mental health services: 1 NHS trust (1 ward) One-to-one observations (substantive staff): Older adult mental health services: 1 NHS trust (2 wards) Adult acute mental health services: 1 NHS trust (2 wards) One-to-one observations (bank & agency staff-cash releasing): Older adult mental health services: 3 NHS trusts (7 wards) Adult acute mental health services: 4 NHS trusts (8 wards) Bedroom self-harm incidents: Older adult mental health services: N/A Adult acute mental health services: 1 NHS trust (2 wards) Bedroom falls at night: Older adult mental health services: 1 NHS trust (2 wards) Adult acute mental health services: N/A A&E visits resulting from bedroom falls at night: Older adult mental health services: 1 NHS trust (2 wards) Adult acute mental health services: N/A Emergency service callouts resulting from bedroom falls at night: Older adult mental health services: 1 NHS trust (2 wards) Adult acute mental health services: N/A **Adult acute inpatient mental health wards clinical inputs** (based on an unpublished VBPMM study in adults, primary data collection from mental health hospital staff interviews, and a published national study of incident reports in the UK) Self-harm incidents: Average number of self-harm incidents per occupied bed day per year: 0.0094 Reduction in self-harm incidents from VBPMM: 44% Staff hours per incident: 9.3 Proportion visiting A&E: 8% **Older adult inpatient mental health wards clinical inputs** (based on a VBPMM study in older adults, primary data collection from mental health hospital staff interviews, and a 2017 NHS Improvement report)**:** Average number of night-time bedroom falls per occupied bed day per year: 0.014 Reduction in night-time bedroom falls from VBPMM: 48% (16% to 88%) Staff hours per night-time bedroom fall: 7.1 Proportion visiting a GP because of a night-time bedroom fall: 60% Proportion of night-time bedroom falls leading to litigation: 0.2% Average A&E visits from night-time bedroom falls per occupied bed day per year: 0.0019 Reduction in A&E visits from night-time bedroom falls with VBPMM: 68% (45% one-sided test) Proportion of patients requiring a hospital stay who visited A&E: 3% Average emergency service visits from night-time bedroom falls per occupied bed day per year: 0.005 Reduction in emergency service visits from night-time bedroom falls with VBPMM: 49% (17% one-sided) **Effect of adopting VBPMM on adult acute mental health inpatient wards:** Metric: number without VBPMM, number with VBPMM, percentage reduction with VBPMM Night-time observations (seconds per observation): 25.8, 14.3, 44.7% One-to-one observations (hours per occupied bed day)—cash releasing: 1.63, 1.20, 26.2% One-to-one observations (hours per occupied bed day)—opportunity cost saving: 0.68, 0.55, 20.4% One-to-one observations (hours per occupied bed day)—total: 2.38, 1.79, 24.4% Self-harm incidents (number per occupied bed day): 0.0094, 0.0052, 44% **Cost impact of adopting VBPMM in adult acute mental health inpatient services:** Metric: NHS and Personal Social Services (PSS), NHS mental health trust Total costs of a VBPMM system: £29,457, £29,457 Reduction in cost of night-time observations: £7380, £7380 Reduction in cost of one-to-one observations—cash releasing: £75,895, £75,895 Reduction in cost of one-to-one observations—opportunity cost saving: £27,038, £27,038 Reduction in cost of self-harm incidents: £12,578, £8449 Total cost without VBPMM: £467,487, £458,104 Total benefits with VBPMM (cost saving excluding cost of VBPMM): £122,891, £118,762 Incremental benefit (benefits-cost of VBPMM): £93,433, £89,305 Incremental benefits (cash releasing only): £46,437, £46,437 Return on investment in relation to total benefits: 3.17, 3.03 Return on investment in relation to cash releasing benefits only: 1.58, 1.58 **Effect of adopting VBPMM on older adult acute inpatient mental health wards** Metric: Number without VBPMM, number with VBPMM, percentage reduction with VBPMM Night-time observations (seconds per observation): 20.25, 10.12, 50% One-to-one observations (hours per occupied bed day)—cash releasing: 1.49, 0.88, 40.4% One-to-one observations (hours per occupied bed day)—opportunity cost saving: 1.11, 0.32, 70.9% One-to-one observations (hours per occupied bed day)—total: 2.61, 1.21, 53.4% Bedroom falls at night (number per occupied bed day): 0.014, 0.0071, 48% A&E visits resulting from bedroom falls at night (number per occupied bed day): 0.0019, 0.0006, 68% Emergency services callouts resulting from bedroom falls at night (per year): 0.005, 0.002, 49% **Cost impact of adopting VBPMM in older adult inpatient mental health services** Metric: NHS and PSS, NHS mental health trust Total costs of a VBPMM system: £29,472, £29,472 Reduction in cost of night-time observations: £29,969, £29,969 Reduction in cost of one-to-one observations—cash releasing: £107,101, £107,101 Reduction in cost of one-to-one observations—opportunity cost saving: £140,335, £140,335 Reduction in cost of bedroom falls at night: £142,948, £17,443 Reduction in cost of A&E visits resulting from bedroom falls at night: £18,308, £0 Reduction in cost of emergency services visits resulting from bedroom falls at night: £4461, N/A Total costs without VBPMM: £854,767, £558,989 Total benefits with VBPMM (cost saving excluding cost of VBPMM): £443,123, £294,848 Incremental benefit (benefits-cost of VBPMM): £413,651, £265,376 Incremental benefits (cash releasing only): £77,628, £77,628 Return on investment in relation to total benefits: 14.04, 9 Return on investment in relation to cash releasing benefits only: 2.63, 2.63	3 low	Yes (in all three papers)
CCTV/video surveillance	Appropriateness (*n* = 2 papers)	• **Krieger et al.** [38]**:** 44% patients reported understanding why they were under video surveillance at the time, 56% reported understanding 4–5 days after [48]. • **Tapp et al. ** [63] conducted a Delphi expert consensus study to try to reach consensus on the elements of high security hospital services that would be essential for the rehabilitation of forensic patients. During round one, 82% of staff and academic experts agreed that “CCTV monitoring should be implemented in the secure environment to reduce institutional incidents”, which met the 80% threshold for consensus. In round three, 62.2% of experts rated CCTV as “Important—the element of care is desirable, but its absence would not have a direct effect on the described outcome [institutional incidents]”. This did not meet the threshold for consensus, which the authors concluded meant that CCTV should not be applied prescriptively in high-secure hospital inpatient services.	2 medium	None
	Adoption (*n* = 3 papers)	• **Nijman et al.** [57]**:** In a survey of 136 acute psychiatric wards in England, 27 (20%) used CCTV for monitoring who was leaving the ward. • **Steinert et al.** [62]: In a survey of psychiatric hospitals in Germany, in general psychiatry and addictions, 15.9% respondents used video monitoring during mechanical restraint. • **Peek-Asa et al.** [59]: “Surveillance cameras and/or mirrors” were implemented by 90.6% (48/53) of psychiatric inpatient facilities in California, and 100% (30/30) in New Jersey (*p* = 0.08).	2 high; 1 low [61]	None
	Penetration (*n* = 1 paper)	• **Krieger et al.** [38] found that 9.4% of patients in their current admission to one of three PICUs in Germany had been monitored via video.	Medium	None
Body-worn cameras (BWCs)	Adoption (*n* = 1 paper)	• **Foye et al.** [52] reported that: • Out of 543 total shift reports completed, BWCs use was reported 50 times, meaning they were used on less than 10% of shifts overall. 78% of these uses were on the acute inpatient ward; only 22% were on the PICU. • There was a moderate positive correlation between BWC use and verbal aggression (r = 0.37, *p* < 0.001), indicating that BWCs were more likely to be used in incidents involving verbal aggression, which do not tend to be documented in Datix. • BWC use was also moderately positively correlated with physical restraint (*r* = 0.31, *p* < 0.001), indicating that BWCs were also more likely to be used alongside physical restraint.	Low	None
	Fidelity (*n* = 2 papers)	• **Hardy et al.** [55] reported that 68% of patients were aware some nurses were wearing BWCs. The patients who said they had not been made aware were from three of the wards, with half from one ward. • **Foye et al.** [52] reported that out of the 50 uses of BWCs during the pilot, the majority of the time, BWCs were activated with a warning (*n* = 30, 60% of activations). 19/50 times (38%) the BWC was deployed with a warning but not activated, and there was only one instance (2%) where the camera was activated without warning.	2 low	None
	Feasibility (*n* = 1 paper)	• **Hardy et al.** [55]**:** • Most (79%) of staff who did *not* wear BWCs reported observing no operational difficulties. • 64% of 39 staff who *did* wear BWCs, and 69% of 23 staff who did *not* wear BWCs reported observing no practical difficulties. The remainder said they were minor and easily resolved, but 9% of staff who did *not* wear BWCs reported that the wearer needed assistance to continue to use the camera. • The Trust’s IT department was not asked to help with any problems during the pilot. There a few minor technical issues reported during the pilot, and these were dealt with by the clinical staff trained to be BWC administrators. • No information governance concerns were raised. The BWC technical/operational difficulties described included: • Difficulties setting up the software • Difficulties connecting to Calla’s web servers • Difficulties securely attaching the BWC • The camera switching on if knocked • Problems switching the camera on/off • The camera not turning on or recording (though this was fixed quickly when reported) • Difficulty wearing the harness over a coat or jacket • Having to take the harness off fully to remove a fleece • The harness smelling (and the wash routine to address this weakening the elastic) • Staff were not taking them back to the docking station after use • Staff difficulties adjusting the harness • BWCs not turning on again after the first monthly generator test—they had to be disconnected and then re-docked.	Low	None
	Costs (*n* = 1 paper)	• **Hardy et al. **[55]**:** Set-up costs: • deliver and attend training, and to create and agree policies: cost of this not specified but stated that time spent on training was 90 min for nine trainers who between them trained 25 staff for a 90-min period. One senior member of staff wrote the policy, which took 3 h. • IT costs: The IT technician spent 48.5 h to set up the service and deal with any problems (cost of this not specified). • Cost of cameras: BWCs and related equipment were provided free of charge for this project. The costs to purchase were: camera and software £6540; accessories £1109. Continuing costs: • Staff time to upload and review recordings (3 h/week from a senior Prevention and Management of Violence and Aggression (PMVA) team member). • Staff costs sorting out problems with cameras (3 h/week from a junior PMVA team member and 1 h/week from a senior PMVA team member). • Storage (provided free of charge for this project) would have cost £569 for the 3-month period.	Low	None
GPS electronic monitoring (EM)	Feasibility(*n* = 1 paper)	• **Tully et al.** [65]**:** did not explicitly report on feasibility but stated that the technology was still in use at the time of publication.	Low	
	Fidelity(*n* = 1 paper)	• **Tully et al.** [65]**:** The technology was used in the way it was intended (in the early stages of a patient being granted leave or transitioning from escorted to unescorted leave). It was only used immediately before discharge in a minority of cases. No data were provided in the paper to support these claims.	Low	None
	Costs(*n* = 2 papers)	• **Murphy et al. **[56] reported the following electronic monitoring costs: • Total electronic monitoring cost over the 3-month study period for 121 devices: £34,653. • Equates to an average electronic monitoring cost per patient of £286. • Total cost per patient (taking into account electronic monitoring costs, staff costs, leave violation costs) was £195,703 overall in the 3-month study period (equivalent to an average of £1617 per patient). Figures these calculations were based on: • Hourly cost of escorting staff: £59 • Annual electronic monitoring contract costs: £114,336 for up to 70 devices • Cost of additional devices: £119/device • Leave violation costs, taking into account factors such as length of violation, whether police were contacted or involved, whether the Ministry of Justice was contacted, any media reports on local or national news, drug/alcohol use or any offences committed during leave (costs were not reported). • **Tully et al.** [65] : Each GPS electronic monitoring device in this study cost £133.	1 medium; 1 low	None
Wearable sensors	Appropriateness(*n* = 1 paper)	• **Tron et al.** [64] reported that: • Movement features detected by the smartwatch during the “free time” window (4–5 pm) were the most effective in explaining variance in patients’ scores on all factors of the Positive and Negative Syndrome Scale. • Combining data from all time windows (free time, lunch, occupational therapy, full day and full night windows) resulted in substantially higher explained variance than any of the individual windows alone for all factors. • They also reported a case where a patient’s step count increased during a period where their medication dosage significantly changed.	Low	None

Acronyms: *A&E* Accident and Emergency, *BWCs* body-worn cameras, *CCTV* closed circuit television, *EM* Electronic Monitoring, *GPS* Global Positioning System, *IT* Information Technology, *MMAT* Mixed Methods Appraisal Tool, *PICU* Psychiatric Intensive Care Unit, *PMVA* Prevention and Management of Violence and Aggression, *PSS* Personal Social Services, *PSSRU* Personal Social Services Research Unit, *ROI* Return on Investment

#### Vision-Based Patient Monitoring and Management (VBPMM)

##### Description of implementation

Nine studies explored VBPMM [32, 39–43, 45–47]. All were UK-based and utilised Oxevision (a VBPMM device by Oxehealth). Eight of the nine studies reported conflicts of interest [39–43, 45–47]. All studies were rated as low quality except one which was rated high quality [32]. This high-quality study was the only VBPMM paper which did not report any conflicts of interest [32]. Inpatient settings included acute wards [41, 43, 45, 47], older adult inpatient mental health wards [41], psychiatric intensive care units [40, 42, 46] and a high secure forensic inpatient service [32]. VBPMM was used in patients’ bedrooms in all but one study, where it was used in a seclusion room [40].

VBPMM involves an anti-ligature, wall-mounted system equipped with an infrared-sensitive camera (a Class IIa medical device), also referred to as an “optical sensor”, which remotely monitors patients’ pulse and breathing rate at regular intervals [41, 45]. It also tracks patients’ movements, generating location and activity-based alerts [43]. Video can be viewed by staff for up to 15 s when taking vital sign measurements or responding to an alert [41, 46]. In the latter case, only blurred video is available [41, 46]. Dewa et al. [32] states that de-pixellated video can “only be viewed with express permission in exceptional circumstances” (e.g. if there is potential risk to the patient), though it did not state who provides permission. The VBPMM system can be accessed via monitors in the nurses’ station and portable tablets [43]. It differs from CCTV in that it has additional physical health monitoring functions and video stream viewing is intermittent “on-demand” rather than continuous observation [45, 46].

Ndebele et al. [45] described how consent for VBPMM use was sought from patients, or from a suitable consultee, such as their carer or the ward’s consultant psychiatrist, in cases where patients lacked capacity to consent. If consent was not given, the system remained switched off in the patient’s bedroom for the duration of their stay. If patients who lacked capacity initially later regained capacity, consent was then sought from them. Malcolm et al. [42] also stated that patients can request for VBPMM to be turned off. The remaining papers did not describe patients being able to opt-in or out of VBPMM use.

##### Stated aims of the technology

Reported aims of VBPMM include as follows: reducing staff disturbance to patients by enabling less intrusive observations [32, 47], allowing staff to respond to patient needs more quickly and efficiently [42, 43], improving safety and quality of care [41, 42], aiding monitoring of self-harm risks [45], monitoring early signs of agitation to enable early intervention for aggression [46], preventing incidents [32, 46], supporting care-planning [39], supporting compassionate and dignified care [39, 43] and reducing NHS mental health care costs. VBPMM is reportedly intended as an adjunct to usual care, not as a replacement for therapeutic interactions or physical care [39, 43, 47]. However, it is unclear how this adjunctive role is envisioned alongside the stated aim of cost reduction.

##### Lived experience involvement in implementation

Three out of nine papers reported lived experience involvement in VBPMM implementation [39, 40, 47]. This included a pre-implementation patient focus group [40] and meetings with former patients, relatives and nursing staff [47]. The Oxehealth report [39] stated that as an organisation, they have continuous patient and caregiver involvement throughout the implementation process. These descriptions of lived experience involvement lacked methodological detail.

##### Implementation outcomes

Five studies reported VBPMM implementation outcomes [41–43, 45, 47]. Barrera et al. [47] reported high fidelity, with no significant gaps in VBPPM use and staff observations being conducted as required, and high penetration, stating that the sensors appeared to be embedded in the ward’s day-to-day clinical practice. In terms of feasibility, Ndebele et al. [45] reported VBPMM consent rates of 67 and 76% on a female and male acute ward, respectively. It was not clear whether any consenting patients later withdrew consent, and whether these figures capture those individuals.

Three studies [41–43] used a cost calculator approach to estimate the costs of implementing VBPMM in addition to standard care, compared to standard care alone. Malcolm et al. [42] calculated that if VBPMM were implemented in addition to standard care for adults admitted to PICUs across England, the total costs per year would be as follows: £10,926 (GBP) per patient, £228 per occupied bed day, £897,907 per average sized ward and £68,839,567 per year to the NHS in total. These calculations were based on estimates that VBPMM would cost £319 per patient (including annual licence fees, installation and cabling and staff training costs), and that the costs per patient would be £158 for night-time observational hours, £9943 for one-to-one observational hours, £167 for assaults and £338 for rapid tranquillisations. A full breakdown of costs and how they were calculated is available in Table 2.

Malcolm et al. [43] estimated that if VBPMM were implemented in addition to standard care for adults admitted to acute mental health wards across England, the total costs per year would be £1879 per patient, £39 per occupied bed day, £205,849 per average sized ward and £153,872,139 per year to the NHS in total. This was based this on estimates that VBPMM would cost £242 per patient (including licence fees, annualised installation and cabling and staff training costs) and that it would cost per patient £63 for night-time observations, £1444 for one-to-one observations and £129 for self-harm incidents. A full breakdown of these costs and how they were calculated is available in Table 2.

From figures provided by Buckley et al. [41], it can be calculated that they estimated the annual cost of an average-sized acute adult mental health inpatient ward with VBPMM in England (with 90% occupancy) to be approximately £374,054 from an NHS and Personal Social Services (PSS) perspective, and £368,799 from an NHS mental health trust perspective. They estimated the annual cost of an average-sized older adult mental health inpatient ward in England (with 90% occupancy) with VBPMM to be approximately £441,116 (NHS & PSS perspective) or £293,613 (NHS mental health trust perspective). They estimated VBPMM costs to be £29,457 on an average-sized acute adult inpatient mental health ward, and £29,472 on an average-sized older adult inpatient mental health ward, including annual licencing fees, annualised installation and cabling fees and staff training costs. A full breakdown of the costs included in calculations in this study is available in Table 2.

#### Closed circuit television (CCTV)/video surveillance

##### Description of implementation

Thirteen studies explored CCTV/video surveillance [38, 48–50, 57, 59–63, 66, 67, 69]. No studies declared conflicts of interest, seven studies were rated as high quality [49, 50, 57, 60–62, 69], three were rated medium quality [38, 63, 67] and three low quality [48, 59, 66]. These studies were based in the UK (*n* = 5), Germany (*n* = 2), Australia (*n* = 1), Ireland (*n* = 1), Finland (*n* = 1), USA (*n* = 1), Malaysia (*n* = 1) and one study recruited experts from a range of countries. CCTV/video surveillance had been implemented in acute wards [49, 61], PICUs [38] and low-secure [67], medium-secure [60] and high-secure forensic wards [63]. Curtis et al. [49] described the setting as an inpatient psychiatric facility with beds for acute psychiatric conditions, geriatric conditions, learning difficulties and forensic cases. In six papers, the type of inpatient ward was not specified [48, 50, 59, 62, 66, 69]. Within wards, CCTV was described as being implemented in communal areas (e.g. ward corridors, exit doors), patients’ bedrooms [67] and seclusion rooms [49, 50, 57, 66]. Some specified that it was not used in private areas such as patient bedrooms [49, 50] or bathrooms [50].

##### Stated aims of the technology

The functions of CCTV/video surveillance described in the papers included monitoring patient behaviour [49, 57, 62, 69] and staff behaviour [49], monitoring who is leaving the ward [57], monitoring safety during mechanical restraint [62], reducing institutional incidents [63] and preventing violence [62].

##### Lived experience involvement in implementation

None reported in the included papers.

##### Implementation outcomes

Five papers reported CCTV/video surveillance implementation outcomes [38, 57, 59, 62, 63]. Krieger et al. [38] reported that only 44% of patients understood why they were under surveillance at the time, and only 56% understood 4–5 days after surveillance ended. Adoption rates varied between studies (from 15.9 to 100% in different locations across the USA, UK and Germany) [57, 59, 62]. In terms of penetration, Krieger et al. [38] reported that 9.4% patients in three PICUs in Germany had been monitored via video, though it was unclear whether all the PICUs had video surveillance technology and its location on the wards.

In terms of appropriateness, Tapp et al. [63] conducted a Delphi expert consensus study to try to reach consensus on the elements of high-security hospital services that would be essential for the rehabilitation of forensic patients. During round one, 82% of staff and academic experts agreed that “CCTV monitoring should be implemented in the secure environment to reduce institutional incidents”, which met the 80% threshold for consensus. In round three, 62.2% of experts rated CCTV as “Important – the element of care is desirable, but its absence would not have a direct effect on the described outcome [institutional incidents]”. This did not meet the threshold for consensus, which the authors concluded meant that CCTV should not be applied prescriptively in high-secure hospital inpatient services.

#### Body-worn cameras (BWCs)

##### Description of implementation

BWCs were investigated in six UK-based studies, two of which were rated high quality [51, 68], one medium quality [54] and three low quality [44, 52, 55]. Conflicts of interest were reported in one study [44]. In four studies, the brand was Calla [44, 51, 52, 55]. Brands were not specified in the other two studies. Inpatient mental health settings included acute wards, low-secure, medium and medium enhanced forensic wards, recovery wards, PICUs and a health-based place of safety room at a psychiatric hospital. Hakimzada et al. [54] explored staff perceptions of BWCs in inpatient settings where BWCs had not been implemented, including acute wards, secure wards and a PICU.

BWCs are recording devices worn by trained staff in inpatient settings to document interactions between staff and patients via audio and video recordings [52]. They are manually activated by staff at their discretion. This may generally be signalled by a red flashing light and audible beep, with staff advised to inform patients before recording [54]. In Hardy et al. [55], staff were trained to explain to staff and patients that the camera was for safety, to narrate their actions and intentions to the camera, and inform patients if they stop recording due to it exacerbating the situation. Staff could turn the camera around to record sound only if necessary [55].

BWC footage access was protected by a PIN to prevent data retrieval if the camera was misplaced [55]. In Hardy et al.’s [55] study, BWCs were docked, recharged and data uploaded to a secure cloud via computer in the reception area at the end of each shift. This secure cloud was provided and administered by the BWC manufacturer. Footage is kept for a fixed length of time before being automatically deleted, unless required for a specific purpose, e.g. internal investigation [44].

Methods of informing patients of BWCs were reported in one study and included displaying information posters in high visibility areas on wards, providing written information, and by staff verbally informing patients about them on admission, in morning meetings, patient experience groups and community meetings [55].

Hardy et al. [55] stated that preparing for BWC implementation involved establishing the necessary policies, IT infrastructure and information governance compliance—e.g. completing a full privacy impact assessment and self-assessment tool from the surveillance camera commissioner. Patients and visitors were informed, and training was delivered to staff by the BWC supplier, which was then cascaded to other ward staff. Certain staff members also received specific training to become administrators [55].

Foye et al. [52] described how, before implementing BWCs, the NHS trust piloting them carried out extensive preparatory work, including directorate and senior management-level planning, consultations with individuals with lived experience, and the development of relevant policies and protocols which incorporated a human rights assessment and legal review. BWC training was provided to staff by the BWC manufacturer.

##### Stated aims of the technology

The aims of BWCs were described as increasing transparency; resolving incidents and complaints by providing accurate incident records; improving staff performance by providing footage for training and monitoring; improving staff conduct and patient behaviour; preventing incidents of violence and aggression; improving safety and to “counter false allegations” [44, 52, 54, 55, 68].

##### Lived experience involvement in implementation

Foye et al. [52] described how before implementing BWCs, the NHS trust piloting them conducted extensive preparatory work, including lived experience involvement and consultation. There was no lived experience involvement in implementation of BWCs described in the other studies.

##### Implementation outcomes

Two studies, both rated low-quality, reported BWC implementation outcomes [52, 55]. In Hardy et al. [55] most staff reported no operational or practical difficulties with the BWCs. Where difficulties were reported, most were minor and easily resolved. Only 68% of surveyed patients reported that they had been made aware that some nurses were wearing BWCs [55]. Hardy et al. [55] reported the following purchase costs: camera and software (£6,540), accessories (£1,109) and storage (£569) though these were provided free by the BWC manufacturer for the study. It also provided a breakdown of staff requirements (e.g. to deliver and attend training, create policies, provide IT support, to upload and review recordings and sort out problems with cameras) but did not report the associated costs.

In Foye et al.’s study [52], BWC use was reported in 50/543 completed shift reports, indicating that they were used on less than 10% of shifts during the 6-month pilot. Seventy eight percent of uses were on the acute adult inpatient ward, and 22% were on the adult PICU. Most deployments occurred with a warning (*n* = 30, 60% activations). 19/50 (38%) of uses the BWC was deployed with a warning, but not activated, and there was only one occasion (2%) where a BWC was activated without warning. There were moderate positive correlations between BWC use and verbal aggression (*r* = 0.37, *p* < 0.001) and physical restraint (*r* = 0.31, *p* < 0.001).

#### Global Positioning System (GPS) electronic monitoring

##### Description of implementation

Two low-quality papers reported on GPS electronic monitoring [56, 65]. Neither reported conflicts of interest. One study used the brand Buddi Tracker [65]; the brand was unspecified in the other. Both studies were set in UK-based medium-secure inpatient mental health services.

In both studies, GPS electronic monitoring devices were attached to patients’ ankles when they went on leave. They were only used with consenting patients, with the exception of high-risk patients requiring urgent hospital or court transfer. It was unclear whether the use of GPS electronic monitoring in these instances was court-ordered or the result of a clinical decision. Consent rates were not provided in either study. Clinical decisions about the appropriateness of GPS electronic monitoring were made on an individual basis following a specific risk assessment protocol in Murphy et al. [56]. Tully et al. [65] described how it was primarily intended to be used with patients in the early stages of leave, when risk of leave violation is highest.

The “Buddi Tracker” device [65] employs secure straps with anti-tamper features, transmitting location via GPS signals to monitoring software via a mobile phone network. Geographical parameters (“geo-fences”) can be set, allowing inclusion and exclusion zones to be created. If a patient breaches a geo-fence, an alarm goes off which causes the device to vibrate and an alert to be sent through the in-built monitoring software. Information from each device is monitored by a security company. Breaches of agreed terms and conditions trigger a predetermined alert to relevant parties and a risk management plan [65].

##### Stated aims of the technology

GPS electronic monitoring tracks patients on leave, with the aim of preventing leave violations such as absconding or failing to return [56]. It was hypothesised to reduce leave violations, increase overall leave and increase the proportion of unescorted leave [56].

##### Lived experience involvement in implementation

Tully et al. [65] states that the introduction of GPS electronic monitoring was discussed with patients and legal advisors, and consent and information forms were developed. However, there is no methodological detail for patient consultation provided. No lived experience involvement in implementation was reported in Murphy et al. [56].

##### Implementation outcomes

Two papers reported GPS electronic monitoring implementation outcomes [56, 65]. Though Tully et al. [65] did not directly discuss feasibility, the authors did state that the technology was still in use at the time of publication, suggesting evidence of feasibility. Tully et al. [65] also reported high fidelity; the authors claimed it was mostly used in the early stages of patients being granted leave or transitioning from escorted to unescorted leave and was only used immediately before discharge in a minority of cases. However, data were not provided to evidence this claim [65]. Murphy et al. [56] calculated that the total cost of GPS electronic monitoring over the 3-month study period was £34,653, equating to an average cost of £286 per patient. They estimated that the total cost of implementing GPS electronic monitoring over the 3-month study period, taking into account the costs of escorting staff (£59 per hour), technology costs (£114,336 for up to 70 devices, and £199 for each additional device) and leave violation costs (cost breakdown not provided), was an average of £1617 per patient. Tully et al. [65] simply reported that each GPS electronic monitoring device used in their study cost £133.

#### Wearable sensors

##### Description of implementation

Two papers, one rated as low quality [64] and one as high quality [53] examined wearable sensors. Neither reported conflicts of interest.

Tron et al. [64] evaluated the use of GeneActiv smart watches for monitoring movement in patients with psychosis at a psychiatric inpatient facility in Israel. These smart watches were equipped with accelerometers, light and temperature sensors. Medical staff managed their placement and removal and uploaded data from the memory card in the device to a central storage location for analysis.

Greer et al. [53] explored staff’s perceptions of using two different remote monitoring devices to conduct real-time monitoring of patients’ psychophysiological signals to predict aggression. One device (E4, Empatica Srl) is worn around the wrist, and the other (Everion, Biovotion Ltd) is worn around the upper arm. Staff were recruited from a medium-secure forensic inpatient service in the UK and did not have prior experience with these devices.

##### Stated aims of the technology

Tron et al. [64] aimed to use the GeneActiv smartwatch to monitor patient movements and correlate them with mental states to better evaluate schizophrenia symptom severity, characterise schizophrenia subtypes and causes, and personalise treatments. In Greer et al. [53], the aim of the devices was to monitor patients’ physical indicators to predict aggression.

##### Lived experience involvement in implementation

Greer et al. [53] stated that the interview topic guide was informed by consultation with two service user–caregiver advisory groups. No lived experience involvement was reported in Tron et al. [64].

##### Implementation outcomes

Tron et al. [64] reported that movement features detected by smartwatches during the “free time” window (4–5 pm) were the most effective in explaining variance in patients’ scores on factors of the clinician-administered Positive and Negative Syndrome Scale (PANSS). Combining data from all time windows throughout the day resulted in substantially higher explained variance on all PANSS factors. They also reported a case where a patient’s step count increased during a period where their medication dosage changed. They argue that this evidence suggests the potential of using smartwatches for continuous tracking of schizophrenia-related symptoms and patient states in hospital settings.

### Research objective 2: pre-implementation: how are surveillance-based technologies in inpatient mental health settings perceived pre-implementation?

#### Vision-Based Patient Monitoring and Management (VBPMM)

One study explored pre-implementation perceptions of VBPMM [40] (see Table 3 for full results). It reported a conflict of interest and was rated low quality. It reported overall positive pre-implementation staff views of VBPMM and mixed patient views. No papers reported carer views.

**Table 3:** Staff, patient and carer pre-implementation perceptions of Vision-Based Patient Monitoring and Management (VBPMM)

Vision-Based Patient Monitoring & Management (VBPMM)​		Pre-implementation perceptions of technology​	MMAT quality rating​	Conflicts of interest​
**Potential uses or benefits**	Staff 1 paper [40] staff *n* = not reported	• Staff largely felt VBVMM would be a **positive addition.** • Thought it would **help obtain vital signs** when it might otherwise be difficult to, given a patient’s presentation​.	Low	Yes
	Patients 1 paper [40] *n* = 12 patients & a patient representative	• Patients largely felt it would be **positively received**, as it was expected to improve **clinical safety** and **reduce disrupted sleep.** • A separate patient representative felt it would be a **positive addition to the seclusion suite.**	Low	Yes
**Concerns and potential harms**	Patients *n* = 1 paper [40] *n* = 12 patients & a patient representative	• One patient was concerned the camera would **emit damaging “rays”.** • Another patient was **worried the camera would control them** in some way. • Another patient suggested it might **reduce human interaction in seclusion.**	Low	Yes

Acronyms: *MMAT* Mixed Methods Appraisal Tool, *VBPMM* Vision-Based Patient Monitoring and Management

##### Staff

Staff were reported to largely feel that VBPMM could be a positive addition to seclusion rooms, as it could facilitate vital sign monitoring [40].

##### Patients

Some patients felt that VBPMM could improve safety and reduce disrupted sleep, whereas others feared that it would reduce human interaction in seclusion, or that the cameras could control or harm them [40].

#### Closed circuit television (CCTV)/video surveillance

Two papers explored pre-implementation perceptions of CCTV/video surveillance [49, 69] (see Table 4 for full results). Neither paper reported any conflicts of interest, and both were rated high quality. Patient views were mixed, and staff and carer views were negative.

**Table 4:** Staff, patient and carer pre-implementation perceptions of closed circuit television (CCTV)/video surveillance

CCTV/video surveillance		Pre-implementation perceptions of technology	MMAT quality rating	Conflicts of interest
**Potential uses or benefits**	Patients 1 paper [69] *n* = 25	• Four patients said they would be happy to be filmed because they would “**enjoy the attention”.** • Some **comfortable** with monitoring, feeling it would **not impact their daily routines.** • One patient would be happy with CCTV in **any location** on the ward. • Most patients were okay with it being **viewed by clinicians and direct family**, with some limitations.	High	None
**Concerns and potential harms**	Patients 1 paper [69] *n* = 25	• Some only comfortable if **mounted in certain places to protect privacy** (e.g. communal areas, not bedrooms or bathrooms). • One person not okay with CCTV in **any** location on the ward. • Some felt the **cameras should be hidden.** • Some felt monitoring would cause **stress**, make them feel **awkward and uneasy**, and **disturbed** to the point it would **impact their daily routines.** • Some **not okay with family monitoring them** through it. • One mention of consent needed for monitoring.	High	None
	Mixed sample (included staff, patients, carers) 1 paper [49] *n* = not reported	• Apprehension about having CCTV in communal areas.	High	None

Acronyms: *CCTV* closed circuit television (CCTV); *MMAT* Mixed Methods Appraisal Tool

##### Patients

Whilst some patients felt comfortable with the idea of being video monitored, others felt that it would cause them stress and disrupt their daily routines. Privacy concerns led some patients to prefer cameras to be positioned in communal rather than private areas. Patient preferences varied regarding camera visibility and who should be able to view the footage [69].

##### Mixed sample (patients, staff, carers)

Curtis et al. [49] reported apprehension towards the use of CCTV in communal ward spaces amongst a mixed sample of staff, patients and carers.

#### Body-worn cameras (BWCs)

Four studies explored pre-implementation perceptions of BWCs [44, 51, 52, 54] (see Table 5 for full results). One reported a conflict of interest [44]. One paper was rated high quality [51], one medium quality [54] and two low quality [44, 52]. Staff views were mixed. No studies reported pre-implementation carer views.

**Table 5:** Staff, patient and carer pre-implementation perceptions of body-worn cameras (BWCs)

Body-worn cameras (BWCs)		Pre-implementation perceptions of technology	MMAT qualityrating	Conflicts of interest
**Quantitative survey results**	**Staff** 2 papers Hakimzada et al. [54]: *n* = 60 nursing staff Ellis et al. [44]: *n* = 15	Quantitative findings from a survey of nursing staff (*n* = 60) [54] • 30% were neutral when asked if they would support BWC use in mental health settings (most common response) • 45% would feel comfortable wearing a BWC • 61.7% felt wearing a BWC would not deter them from working • 35% felt BWCs would de-escalate violent situations on the ward • 75% were confident in the ability of BWCs to reduce false patient accusations. This item had the lowest negative response (8.3%). • 51.7% agreed BWCs could “resolve violent incidents” • 50% agreed BWCs would put their mind at ease • 55% felt BWCs would cause staff to modify their behaviour • 56.7% agreed there may be ethical issues regarding patients being recorded in compromising situations • 65% agreed there may be ethical issues regarding patient confidentiality	1 × low 1 × medium	1/2 papers reported a conflict of interest
		Quantitative findings from a questionnaire to mental health ward staff (*n* = 15) [44] • 80% thought BWCs would have a positive impact • 86% thought BWCs help reassure both staff and patients • 100% encountered verbal or physical aggression at least once a week • 87% spent a “considerable portion of their time dealing with aggressive behaviour” • 80% said dealing with aggressive behaviour “often gets in the way of doing the job they ought/want to be doing” • 80% said if BWCs could help reduce aggressive behaviour or the time spent dealing with it, “it would have a positive impact on their day-to-day job” • 60% could recall a work incident “where they wished they’d had a body camera”		
**Potential uses or benefits**	**Staff ​** 3 papers [44, 52, 54] Hakimzada et al. [54]: *n* = 60 ​nursing staff Ellis et al. [44]: *n* = not reported Foye et al. [52]: *n* = 16	• Reduce and deal with **false patient accusations.**​ • Enable **accurate, unbiased evidence documentation** of incidents. • Increase **staff and patient safety.**​ • **Reduce violent and aggressive incidents.**​ • Potential use as a **training, learning** or **reflective tool** for staff to **reinforce good practice** and identify faults in staff behaviour​. • Cause patients to **“think before acting”.**​ • **Monitor the interaction** between patients and staff. • **Similar protocols to other security measures** made staff view BWCs **as easier to implement**. • A minority of staff felt BWCs could **improve relationships** by providing **transparency** around staff behaviour and **encouraging positive staff behaviour** and **high-quality care**.	2 × low 1 × medium	1/3 papers reported a conflict of interest
	**Patients** 1 paper Foye et al. [52]: *n* = 5	• May **hold staff to account** for their own behaviours and so improve care, though concern expressed that this effect may **wear off after the first few months** as people may forget about the cameras being there.	Low	None
**Concerns and potential harms**	**Staff ​** 3 papers [51, 52, 54] Hakimzada et al. [54]: *n* = 60 ​nursing staff Foye et al. [52]: *n* = 16 staff Foye et al. [51] *n* = 31 staff	• **Violates patient confidentiality**, which could lead to legal action against Trusts​. • BWCs are intrusive/**violate patient privacy.**​ • Increase patient **paranoia, aggression, annoyance**, make them feel **intimidated.**​ • Particular concerns around potential negative impact on patients with **paranoia**, **psychosis,** and **previous negative experiences of being filmed.** • **Ambivalence** amongst some staff that introducing BWCs would reduce violence and aggression, due to **acceptance of violence and aggression being “part of the job”**. • BWCs could **aggravate violent situations**​ or **instigate incidents.** • Could **negatively impact therapeutic relationships, increase power differentials** between patients and staff**,** and make it **difficult for patients to trust staff.**​ • Could be **unethical.**​ • Could **increase assault against staff**/make staff a target​. • **Issues obtaining patient consent**, and some patients may not understand the rationale for them​. • **Continued information surrounding BWC use** needs to be provided to patients, as their health and capacity may fluctuate during their stay on the ward. • Staff would be **uncomfortable** wearing BWCs​. • Staff may **forget to wear BWCs**, **forget to switch them on**, and **forget to charge them**. • Staff may be **unable to use BWCs correctly**. • Staff may be **less willing to get involved in incidents.** • Staff/patients could **“act”** for the camera. • Patients could **break the BWCs**/use them as a **weapon.** • **Doubts over transparency** of using cameras to collect evidence around incidents as **cameras remain in staff control**, resulting in issues of **bias and power**. • When multiple initiatives and directives were **introduced simultaneously**, staff felt negative towards interventions more generally, viewing BWCs as **“just another thing to do”** which **increased their workload** and **reduced staff enthusiasm** for BWCs. • **Staffing issues,** contributing to **higher workloads, higher rates of bank and agency staff**, and **staff burnout** are barriers to implementing BWCs. • Need for a **continued training approach** as opposed to a one-off session during induction to ensure **changes in personnel, practice and shift patterns** are part of the implementation process.	1 × high 1 × medium 1 × low	None
	**Patients** 1 paper Foye et al. [52]: *n* = 5 patients	• Concerns about BWCs being used to collate evidence relating to incidents as cameras remain in **staff control**, creating issues of **bias and power**—more so than CCTV, which was viewed as more neutral. • Some felt BWCs **do not reduce incidents** as some patients are **too acutely unwell to have insight** into their own actions or those of staff activating the cameras. • Perception of BWCs as an intervention for staff intervention and safety, thereby **increasing the culture of “them and us”** and creating patient resistance towards BWC use. • Potential for resistance from patients with **previous negative experiences** of police use of BWCs or **mistrust in staff**. • **Staffing issues,** contributing to **higher workloads, higher rates of bank and agency staff**, and **staff burnout** are barriers to implementing BWCs. • BWCs **do not address wider systemic issues** contributing to violence and aggression.	Low	None

*Acronyms*: *BWCs* body-worn cameras, *MMAT* Mixed Methods Appraisal Tool

##### Staff

There were mixed views amongst nursing staff about whether they would feel comfortable wearing a BWC, whether it would deter them from working, modify staff behaviour or put their minds at ease. Some felt that BWCs could be a useful training and reflection tool, helping to reinforce good practice and identify faults in staff behaviour. However, others thought they may make staff less willing to get involved in incidents, or that staff and patients may “act” for the camera. Some staff felt that footage from BWCs could provide accurate, unbiased documentation of incidents, and most felt that they would reduce “false patient accusations”. However, some questioned the transparency of BWC recordings, highlighting concerns about bias and power dynamics due to the cameras being controlled by staff.

Some staff believed that BWCs could improve staff and patient safety and help reduce and de-escalate conflict and violent incidents, and so reduce constraints on patients. However, others thought they could increase and exacerbate violent and aggressive situations. Staff expressed concerns about the cameras intimidating patients, and increasing paranoia, particularly for patients experiencing psychosis or with previous negative experiences of being filmed. A minority thought BWCs might enhance staff-patient relationships, whilst others believed they could harm therapeutic relationships, increase power differentials between patients and staff and undermine patient trust. Furthermore, ethical concerns were raised by some staff regarding the difficulty of obtaining informed consent, and the potential of BWCs to violate patients’ privacy and confidentiality.

On a practical level, some staff believed that having similar protocols to other safety measures, such as personal alarms, could make BWC implementation easier. However, concerns were also raised about BWCs increasing workloads, staff forgetting to wear, activate and charge the cameras, and fears that BWCs could be broken and used as weapons by patients. Staffing issues, such as reliance on bank and agency staff, were considered a barrier to effective BWC implementation. Staff emphasised the need for continued BWC training, rather than a one-off training session during induction, to ensure changes in personnel, practice and shift patterns are part of the implementation process [44, 51, 52, 54].

##### Patients

Some patients believed BWCs could hold staff accountable and improve care, though worried that this effect would wear off after a few months as people become accustomed to the cameras. Concerns were raised about the transparency of BWC recordings, as the cameras are controlled by staff, creating issues of bias and power. Some patients felt that BWCs would not reduce incidents due to some patients being too acutely unwell to have insight into their actions or those of staff activating the cameras. Others doubted their effectiveness in reducing incidents since they do not address the wider systemic factors contributing to violence and aggression. Some patients viewed BWCs as being an intervention primarily for staff safety and benefit. They viewed them as increasing a culture of “them and us”, leading to patient resistance towards BWC use, especially amongst people with mistrust towards staff or negative experiences of BWC use by police. Like staff, patients viewed staffing issues and burnout as barriers to effective BWC implementation [52].

#### Global Positioning System (GPS) electronic monitoring

No papers reported on staff, patient or carer pre-implementation perceptions of GPS electronic monitoring.

#### Wearable sensors

One paper explored pre-implementation perceptions of wearable sensors [53] (see Table 6 for full results). It did not report any conflicts of interest and was rated high quality. Staff views of wearable sensors were mixed. No studies reported patient or family/carer views.

**Table 6:** Staff, patient and carer pre-implementation perceptions of wearable sensors

Wearable sensors		Pre-implementation perceptions of technology	MMAT quality rating​	Conflicts of interest
Potential uses or benefits	Staff 1 paper [53] *n* = 25 nurses	• Could help **monitor risk** and so **prevent situations escalating**, **reducing violent incidents.** • Provides **information** which patients may otherwise not express or which may not be observable by staff. • Could **foster self-awareness** amongst patients. • **Facilitates less obtrusive monitoring** without the need for physical contact. • Factors that could increase patient willingness to engage could include stylish design and having clear benefits to wearing the device (e.g. if it affected their leave status).	High	None
Concerns and potential harms	Staff 1 paper [53] *n* = 25 nurses	• Device could be used as a **ligature** due to elastic armband. • Device could be used as a **weapon** to cause** harm to self or others.** • Could **exacerbate patient paranoia.** • Concerns about** data security and patient confidentiality.** • Could **add to staff’s workloads **(e.g. if need to manually upload/analyse data, monitoring patient use of the technology, or if checklists accompany them). • Patients’ willingness to use the technology may **change depending on their mental state.**	High	None

Acronyms: *MMAT* Mixed Methods Appraisal Tool

##### Staff

Staff recognised wearable sensors’ potential for facilitating less obtrusive monitoring, increasing patients’ self-awareness and providing information that may not otherwise be shared with staff. Some also felt that they could aid risk-monitoring, reduce violent incidents and prevent situations from escalating. However, concerns included patients misusing them as ligatures or weapons, exacerbating patient paranoia, data security and patient confidentiality issues, fluctuating patient willingness to use them and increased workload for staff.

### Research objective 2: post-implementation: How are surveillance-based technologies in inpatient mental health settings experienced post-implementation?

#### Vision-Based Patient Monitoring and Management (VBPMM)

Six papers explored post-implementation experiences of VBPMM [32, 39, 40, 45–47] (see Table 7 for full results). Five of these studies reported conflicts of interest [39, 40, 45–47]. Five were rated low quality [39, 40, 45–47], one was rated high quality [32]. Experiences of patients, staff and carers were mixed.

**Table 7:** Staff, patient and carer post-implementation experiences of Vision-Based Patient Monitoring and Management (VBPMM)

Vision-Based Patient Monitoring & Management (VBPMM)​		Post-implementation experiences of surveillance​	MMAT quality rating​	Conflicts of interest​
**Perceived benefits​**	Staff (*n* = 5 papers)​ [32, 40, 45–47]	• Positive effect on **patients’ sleep​.** • Enables **less intrusive monitoring,** reducing the risk of aggressive behaviour escalating, and **improving patient/staff relationships.** • **Observations easier and quicker** for staff​. • Perceived **reduction in verbal and physical aggression.**​ • Knowing location of patients thought to **help staff manage large wards.** • Graphical reports provide an overview a patients’ day and/or night level of activities, providing a **snapshot of acuity** which can be useful in **staff handovers** and help staff **better plan daytime activities**. • Perceived **improvement to patients’ privacy and dignity** when compared to in person observation​. • Provides patients with a **greater sense of agency** for their safety. • Technology helps **identify, prevent and mitigate incidents​**, **reducing restrictive interventions.** • Leads to **better care** for patients​. • Improved **staff and patient safety.** • Improved **assurance for staff managing risk.** • Can serve as **an extra safety measure** when staff were unable to perform physical checks on a patient (e.g. if they were behaving aggressively). • Improved physical health monitoring **aiding clinical decision making​.**	4 × low 1 × high	4/5 papers reported a conflict of interest
	Patients (*n* = 3 papers)​ [32, 39, 40]	• **Feeling safer** as monitoring leads to staff helping quicker if their health worsens​. • Technology **aids independence** from staff​. • **Better nights’ sleep** with remote monitoring (as physical checks disturbed sleep)​. • Monitoring in seclusion aided **feeling connected to others.**​ • Some patients feel **indifferent** about the technology’s use, for example, over time forgetting that it was there, paying less attention to it, and accepting that it was there to stay.	2 × low 1 × high​	2/3 papers reported a conflict of interest
	Carers (*n* = 1 paper [40])	• Carers **had mostly positive perceptions** of monitoring.	Low​	Yes
**Negative impacts, effects and harms**	Staff (*n* = 1 paper [32])​	• **Technological glitches** (e.g. poor Wi-Fi, signal issues, poor readings of patient activity)​. • **Security concerns**; **data protection** and **physical concerns** about the device e.g. concerns about patients accessing VBPMM data via the code on the back of staff’s iPads. • **Lack of trust** in the technology's **accuracy.**​ • **Insufficient training** to be able to use the technology correctly, and **issues with staff ability** to use the technology​. • Technology **not a replacement for standard care** and physical observations​. • Negative effect on patients’ **privacy** including **ethical concerns** regarding watching patients.	High	None
	**Patients** (*n* = 1 paper [32])	• **Lack of privacy and dignity** felt when monitored​. • Concerns regarding the **impact on human rights.** • Feelings of **embarrassment, distress and paranoia** regarding being watched (particularly around getting undressed)​. • **Lack of choice** or say about the use of the technology​. • **Less trust in staff** and **impact on relationships** with staff​. • **Increased power imbalance** between staff and patients​. • **Lack of communication** about the technology, including **inaccuracies in explanations. ** •Negative perceptions more common amongst patients who had spent less time in hospital.	High	None
	Carers (*n* = 1 paper [40])​	• Concerns regarding the** negative impact on quality of care.**	Low	Yes

*Acronyms*: *MMAT* Mixed Methods Appraisal Tool, *VBPMM* Vision-Based Patient Monitoring and Management

##### Staff

Benefits of VBPMM perceived by staff included less intrusive monitoring, which could improve patients’ sleep, and enhanced safety for staff and patients, through better physical health monitoring, reduced patient aggression and more effective prevention and mitigation of incidents. Some staff also believed that VBPMM gave patients a greater sense of agency over their own safety, could reduce use of restrictive practices and contributed to improved staff-patient relationships. There were mixed perspectives on its impact on patients’ privacy. Staff also flagged concerns about technological issues (e.g. poor Wi-Fi), incorrect use of the technology, insufficient staff training and doubts about its accuracy. Some felt VBPMM should not replace standard care and physical observations [32, 40, 45–47].

##### Patients

Some patients also felt that VBPMM improved patient safety and sleep. Other benefits reported by patients included increased independence from staff and a greater sense of connection in seclusion. However, patients also raised ethical concerns about VBPMM’s negative impact on their privacy, dignity and human rights. They cautioned about how being monitored can cause distress, exacerbate power imbalances and damage trust between patients and staff. Concerns were also raised about a lack of patient choice, and inadequate or inaccurate communication from staff regarding VBPMM [32, 39, 40].

##### Carers

One paper reported that carers had mostly positive perceptions of VBPMM, but some had concerns about a negative impact on care quality [40].

#### Closed circuit television (CCTV)/video surveillance

Five papers explored post-implementation experiences of CCTV/video surveillance [49, 50, 60, 66, 67] (see Table 8 for full results). None of these studies reported any conflicts of interest. Three were rated high quality [49, 50, 60], one medium quality [67] and one low quality [66]. Three studies explored experiences of CCTV/video surveillance in communal ward areas [49, 50, 66], one in a seclusion room [60] and one in patients’ bedrooms [67].

**Table 8:** Staff, patient and carer post-implementation experiences of closed circuit television (CCTV)/video surveillance

CCTV/video surveillance		Post-implementation experiences of surveillance​	MMAT quality rating​	Conflicts of interest​
**Perceived benefits​**	Staff (*n* = 3 papers)​ [49, 50, 67]	• Staff became **accustomed** to CCTV in communal spaces​. • Staff found CCTV reassuring and useful for **monitoring and preventing absconding, self-harm and violent behaviour​.** • Video footage as **evidence against allegations** (useful in the aftermath of incidents for establishing responsibility)​. • CCTV felt by some to be an **effective means for observations during the night.**​ • CCTV in bedrooms **less disruptive to patients’ sleep** compared to physical observations. ​ • **Improved staff safety** as remote monitoring allows them to assess behaviour.	1 × medium​ 2 × high	None
	Patients (*n* = 5 papers) [49, 50, 60, 66, 67]	• Patients became **accustomed** to CCTV in communal spaces and found it acceptable​. • CCTV in communal spaces **did not appear to affect patients’ use of these spaces.** • Some patients did **not find CCTV intrusive.**​ • Observation via CCTV useful for **early recognition/detection of emergencies** and **faster intervention** from staff (e.g. self-harm or medical emergencies)​. • Patients felt CCTV helped **ensure patient safety.**​ • Remote monitoring helps **reduce disturbance** at night​. • More appropriate for those who **are very unwell** and on a lot of medication (e.g. to ensure regular monitoring to avoid physical health emergencies)​. • Improves patient safety as it **deters other patients from violence and rule breaking.**​ • Feel safe as images and footage are **confidential.**	1 × low​ 1 × medium​ 3 × high	None
**Negative impacts, effects and harms**	Staff (*n* = 3 papers) [49, 60, 67]	• Concerns about impact on **privacy, dignity and human rights.** • Concerns that **staff behaviour is under scrutiny​.** • **Doubts if CCTV is a good substitute for the presence of a nurse in person**—use of faceless technology loses the therapeutic engagement element of observations and care​. • Useful for **the aftermath of incidents** but **not preventing them**, therefore does not make staff feel safer. • Concern that cameras and monitoring made patients **more paranoid and unwell**, and increased patients feeling of **unease​.** • Experiences of patients being monitored by CCTV **outside of designated hours and without consent.** • Remote observations **removed human connection** thus had an impact on **quality of care** and negative effect on **staff-patient relationships​.** • Technology was **unreliable** and **poor-quality images** meant physical observations were needed​. • Staff reported **lack of confidence** using the technology, with bank staff unsure how to use it.	1 × medium​ 2 × high	None
	Patients (*n* = 3 papers) [60, 66, 67]	• **Mixed views** with TV monitoring slightly more negative after implementation on one ward, and slightly more positive on the other​. • Feelings of **lack of control** over observation when via CCTV​. • Concerns about **security and privacy**, increased by poor communication about the technology, and with preference for pixelated images​. • Remote observations removed **human connection** impacting on **communication and relationships with staff​.** • Feeling** unable to relax** when being watched due to intrusion in **personal space.​** • Concerns that **traditional observations will be overlooked** when technology is present​.	1 × low​ 1 × medium​ 1 × high	None
	Carers (*n* = 1 paper [49])	• Concern that staff will not always be watching the CCTV monitor so might **miss things.**	High	None

*Acronyms*: *CCTV* closed circuit television, *MMAT* Mixed Methods Appraisal Tool

##### Staff

Staff’s experiences of CCTV in communal spaces varied [49, 50, 66]. Some identified benefits including improved staff and patient safety, monitoring of self-harm, violence and absconding. However, others doubted its ability to control behaviour or prevent incidents. Some saw value in using CCTV to provide evidence to investigate incidents and allegations and felt it could be used to scrutinise staff behaviour. Ethical concerns were raised about its impact on patients’ privacy, on dignity and human rights and on therapeutic engagement. Some staff felt CCTV should not be used as a substitute for in-person care [49].

Staff’s views of CCTV use in patients’ bedrooms at night were also mixed [49, 50, 67]. Perceived benefits included improved monitoring of patients, enhanced staff safety and reduced disruption of patients’ sleep compared to physical checks. Some staff felt they could rely on CCTV for patient observation, whereas others emphasised the importance of still conducing physical checks. Some staff raised concerns about negative impacts of CCTV in patients’ bedrooms on privacy, increased patient distress and paranoia and reduced opportunities for therapeutic engagement. There were also reports of staff feeling uncertain about how to use the technology, using it incorrectly, finding it unreliable and it producing low-quality images [67].

##### Patients

Patients had mixed views on CCTV monitoring in communal areas. Some felt it enhanced staff and patient safety, whilst others considered it an invasion of privacy. CCTV use in communal areas did not appear to affect patients’ use of these spaces [50]. In seclusion rooms, some patients believed CCTV could aid staff observations, prevent self-harm, help recognise emergencies and foster a sense of safety. However, concerns included a lack of control, privacy issues and security concerns, worsened by poor communication about the technology [60].

Regarding CCTV use in patients’ bedrooms at night, some patients found it enhanced their sense of safety, for example by deterring other patients from rule-breaking or stealing property. Some considered it less invasive and disruptive to sleep than physical checks since it reduced staff movement and the frequency of staff entering bedrooms for checks. However, others felt it was intrusive, impeded relaxation, negatively impacted therapeutic relationships with staff, and feared that it could result in traditional observations being neglected. Misunderstandings amongst patients about how and when CCTV was being used were reported, and there were also instances where patients were video monitored in their bedrooms outside of designated times or without consent [67].

##### Carers

One study reported that carers had concerns staff would not always be monitoring CCTV and so may miss things [49].

### Body-worn cameras (BWCs)

Four papers explored post-implementation experiences of BWCs [51, 52, 55, 68] (see Table 9 for full results). None reported any conflicts of interest. Two were rated high quality [51, 68] and two low quality [52, 55]. Staff and patient experiences were mixed, no carer experiences were reported.

**Table 9:** Staff, patient and carer post-implementation experiences of body-worn cameras (BWCs)

Body-worn cameras (BWCs)		Post-implementation experiences of surveillance	MMAT quality rating	Conflicts of interest
**Perceived benefits**	Staff (*n* = 4 papers) [51, 52, 55, 68]	• **Staff wearing cameras were more positive** than other staff about them; **staff not wearing them had more mixed views.** • Belief or experience that it **prevents violence and aggression.** • Useful as **evidence for complaints/resolving incidents** and can **save time during investigations**. • Considered a useful tool for **prosecution** following incidents of violence. • Staff often felt BWC footage could be used **to protect them against false accusations of misconduct** and so **make staff feel safer.** • **Reassured** in their techniques in restraint when cameras are on. • May reveal when **staff are not behaving professionally**. • Potential use in **training** with some staff expressing a desire to watch footage of incidents during a debrief with a manager to reflect on their own behaviour and consider what they might do differently in future. • Some staff believed BWCs would be useful **for documenting physical restraint and planned interventions**, potentially **reducing restrictive practice** and **increasing physical safety for both staff and patients.** • Staff tended to believe BWCs could make wards a safer place by **improving staff awareness and reflexive practice**, rather than changing patient behaviour. • Staff felt cameras were **small** and **easy to use.** • Staff felt that **not having to upload and manage BWC data** made them **user friendly**. • In one study, most staff felt BWC use had **little direct impact on their daily routines**.	2 × low 2 × high	None
	Patients (*n* = 2 papers [52, 68])	• Many patients expressed feeling unheard, ignored and not believed by staff – BWCs may make patients feel **safer by providing evidence to back up their claims**. • Patients see the potential for BWCs to **protect them from staff misconduct.** • Patients believed BWCs would be useful for **documenting physical restraint and planned interventions**, potentially **reducing restrictive practice** and **increasing physical safety** for both staff and patients. • Patients tended to believe BWCs could make wards a safer place by **improving staff awareness and reflexive practice**, rather than changing patient behaviour.	1 × high 1 × low	None
**Negative impacts, effects and harms**	Staff (*n* = 4 papers) [51, 52, 55, 68]	• **Design issues** negatively impact BWC use and acceptance by staff e.g. some staff found BWCs caused **discomfort** to wear, **pulling on clothing**, **infection control** challenges, and cameras being **easily grabbed, thrown and broken**. • Issues with **staff forgetting to wear cameras**, **forgetting to switch them on** during incidents, and **forgetting to charge them** at the end of the shift, reducing potential use by other staff. • Concerns about **useability** for staff **less confident with technology.** • **Charging** and **storage issues** on busy wards. • Concern footage **only captures from time of arrival, not the build up​.** • **BWC footage is biased** since **staff are in control** of the filming, whereas **CCTV is more impartial.** • Staff **feel watched​.** • Patients appear to feel **intimidated** by the technology. • Some staff feel BWCs **do not prevent violence and aggression** because they treat it as an individual issue **without addressing complex systemic causes**. • Many staff felt that **de-escalation skills, communication and positive relationships** should be **primary tools for violence reduction**, before BWCs. • Potential for BWCs to **increase patient aggression** and **instigate incidents**. • Some felt BWCs **do not reduce incidents** as some patients are **too acutely unwell to have insight** into their own actions or those of staff activating the cameras. • Some staff were concerned that BWCs would be treated as a **substitute for good care or safe staffing**. • BWCs could **negatively impact therapeutic relationships** between patients and staff by increasing a **“them and us” dynamic** due to the power differential of staff controlling cameras. • Some staff raised **ethical and legal questions** about the role **of patient consent** in deciding when, or if, a BWC is turned on. • Some were concerned BWCs would feel like a **punitive measure** that singles out a patient, enhancing existing feelings of **criminalisation** and making the **ward feel less safe**. • Some staff viewed BWC implementation directives as being given by senior management and policy stakeholders with **limited understanding due to a lack of “frontline” mental health service experience,** resulting in a **lack of faith in BWCs**, and feelings that **funds were being misspent**. This was **exacerbated by a lack of explanation or consultation.** • **Lack of consistency** across the country in how BWCs are used. • **Lack of evidence** of BWCs reducing incident rates or severity contributes to **lack of staff buy-in and BWC use.** • **Lack of consistency in staff understanding** of the purpose and processes of using BWCs, contributing to **lack of transparency** and **trust** regarding use of subsequent video footage. • Staff widely felt **BWC training was insufficient** and **overly focused on operational aspects**. Staff emphasised the need for **longer training sessions** covering **ethical considerations, decisions on when or when not to use them, sensitivity** of using cameras in potentially triggering situations, **communication techniques**, and their **impact on therapeutic relationships, treatment and recovery.** • **Inconsistency of training delivered**, with some staff receiving **no or very limited training** and other services delivering a **range of training for all staff members** • Training needs to be delivered on a **rolling basis,** to ensure that bank staff are trained in BWC use. • **Ambivalence** amongst some staff that introducing BWCs would reduce violence and aggression, due to **acceptance of violence and aggression being “part of the job”**. • Incidents can occur **quickly and suddenly**, and the **reactive nature** of the ward environment means staff may react instantly so **are not thinking about new processes** (e.g. turning on BWCs), resulting in **failure to use them**. When staff did remember or had the opportunity to switch the camera on, it was often **too late**. • Perception that BWCs **increase workload negatively impacts acceptability** amongst staff. • View that BWCs do not address **systemic issues contributing to violence and aggression**, and that there is a **need to invest in staff and training,** and **more support for staff,** rather than new technologies, including from management. • **Lack of evidence for the impact** of BWCs a **barrier** to consistency and a national approach to implementation—there is a need for more, independent, in-depth research with a focus on patient care to guide policies. • **Robust governance around data management** is needed to assure patients and carers of their safety and the safety of their data. • **Continued information surrounding BWC use** needs to be provided to patients, as their health and capacity may fluctuate during their stay on the ward. • Concerns that the technology would be **costly**, with **implications for resources**. • Frequent staff movement between wards, changing shift patterns, and use of bank staff increases the **risk of losing BWCs**, leading to **financial costs** and **reduced availability** on the ward. • Some staff **in senior and leadership roles** who were responsible for reviewing recordings mentioned the **additional time and cost** of having to **review footage**. • Concern that BWCs would be used as a **tool to monitor staff behaviour**. • BWCs could act as a **“trigger”** and make patients **more distressed**. • To ensure **privacy and dignity** for patients, there need to be **limits** around the use of BWCs and **flexibility** to not use them.	2 × low​ 2 × high	None
	Patients (*n* = 4 papers [51, 52, 55, 68]	• **Negative impact on relationship between staff and patients** with patients expressing hesitation about speaking with staff members wearing a camera, regardless of whether it is on or off. • Some patients were concerned that BWCs would be treated as a **substitute for good care or safe staffing.** • Some patients raised **ethical and legal questions** about the role of **patient consent** in deciding when, or if, a BWC is turned on. • Some patients were concerned BWCs would feel like a **punitive measure** that singles out a patient, enhancing existing feelings of **criminalisation** and making the **ward feel less safe**. • Increased feeling of **staff having power and control over patients**. • Patients were concerned about being recorded in their **most vulnerable moments** and the impact BWCs might have on their **recovery, dignity and privacy**. • Could negatively impact patients experiencing **paranoia, psychosis** or people with **previous negative experiences of being filmed.** • BWCs could act as a “**trigger”** or make patients **more distressed**. • Some patients felt that **BWC footage is biased** since **staff are in control** of the filming, compared to CCTV which was viewed as more impartial. • Footage can **only be captured when staff are present** at an incident. • **Lack of information about the purpose and processes** related to BWCs, with most patients reporting that they **did not receive information** about them during their admissions and were unaware of their rights. • BWCs **do not address systemic contributors to violence and aggression** on wards. • **Lack of evidence for the impact** of BWCs viewed as a **barrier** to having consistency and a national approach to implementation—there is a need for more, independent, in-depth research with a focus on patient care to guide policies. • Some felt a **national directive** around BWC use is needed due to **inconsistency** in how they are implemented by Trusts. • **Robust governance around data management** is needed to assure patients and carers of their safety and the safety of their data. • **Hardware** needs to be **durable and hardy** to ensure they BWCs and their footage are safe and secure. • Concerns about potential **misuse of footage by staff** members or that patients **may not be able to obtain access to footage** if requested. • **Fears about images appearing on social media** or being used by **government bodies involved in anti-terrorism, asylum applications or welfare benefits**—policies, procedures and protocols are needed before BWCs are implemented to protect vulnerable patients. • **Continued information surrounding BWC use** needs to be provided to patients, as their health and capacity may fluctuate during their stay on the ward. Different mediums are needed to ensure everyone is informed irrespective of their literacy, capacity, or health. • Staff need to be **trained** not only in technological aspects of BWCs, but also about **ethical considerations, decisions on when or when not to use them, sensitivity** of using cameras in potentially triggering situations, **communication techniques**, and their **impact on therapeutic relationships, treatment, and recovery.**	2 × low 2 × high	None
**Neutral views**	Staff (*n* = 2 papers) [51, 52]	• **Policies and governance** should be developed in a **thoughtful and sensitive way** given the complex nature and feelings expressed about BWCs, to ensure they are used **in the right way and for the right reasons**. This includes considering **how, why, what, where and when** they should be implemented, and building an **open culture** about BWCs **from the beginning of the implementation process.** • **Feedback from staff** was considered important to **problem-solve difficulties and concerns** and ensure **continued improvement of BWC implementation.** • **Leadership and management** is needed from the **beginning** of the implementation process to ensure BWCs are implemented in an ethical and appropriate way. • **Patients need to be informed about how, why and when BWCs are used**, and their **rights**. This helps **ensure transparency** and **holds staff to account** for using them correctly. • **Visible policies and procedures** provide **assurance** to patients about why BWCs are being used and how, and to ensure **accountability and transparency.** A **range of methods** of informing patients can be used, including posters, community meetings, and leaflets in admission packs. • **Good technical infrastructure and IT support** are needed to support data management plans and policies underpinning BWC implementation. • Value of **feedback loops** to refine and improve BWCs and their implementation. • **Procedures for reviewing BWC footage varied across Trusts**, with some noting that they needed to review all footage, whereas others intended to only review footage related to incidents. Staffing costs for management of footage were therefore dependent on the nature and use of the footage. • Many felt that BWCs **should not be used in private spaces** (e.g. bedrooms, bathrooms). However, acknowledged that **boundaries are not easy to adhere to** when incidents occur in private spaces. It was therefore felt that there should be a policy of not using BWCs in private spaces unless it was “**really necessary”.** • BWCs need to be **simple and easy to use**, and **not add considerably to staff workloads**. • Importance of **consultation with staff** to **understand challenges** to implementation, gauge levels of “**buy in”**, and inform subsequent training, and to acknowledge staff perspectives, including **accepting** if implementation was **not the right choice** for staff and the trust. • Need to take a **person-centred** approach and weigh up the pros and cons to using BWCs in specific situations—avoid using them when they may have a negative impact.	1 × high 1 × low	None
	Patients (*n* = 1 paper) [51]	• Need for **policies and procedures** to ensure BWCs are used in the **right way for the right reasons**, with **clear boundaries**, and to ensure a **consistent culture and understanding**. • Many patients felt they would be more **comfortable being filmed in communal areas**, but not private areas (e.g. bedrooms, bathrooms). However, acknowledged that boundaries are **not easy to adhere to** when incidents occur in private spaces so there should be a policy of not using BWCs in private spaces unless **“really necessary”**. • Importance of **consultation with patients** to ensure BWC implementation is **sensitive to their needs and concerns**, particularly so when considering people who are **autistic**, experiencing **psychosis**, or have a **history of trauma,** who all may be negatively impacted by BWC presence.	1 × high	None

Acronyms: *BWCs* body-worn cameras, *MMAT* Mixed Methods Appraisal Tool

#### Staff

Benefits of BWCs perceived by staff included reduced violence, aggression and restrictive practices. Some staff felt that they improved safety by improving staff awareness and reflexive practice, rather than changing patient behaviour. Staff identified various uses for BWC footage including: providing evidence to aid incident and complaint resolution (including “false allegations” against staff) and prosecutions; documenting interventions (e.g. physical restraints); and facilitating debriefing and staff training. However, concerns were raised about potential bias, as BWCs remain in staff control and only capture footage from the time of arrival, not the preceding events. Some staff also expressed concern about BWCs being used to monitor staff behaviour.

Some staff were sceptical about the effectiveness of BWCs in reducing violence and aggression. Some staff felt this is because they do not address underlying systemic causes, and because some patients are too acutely unwell to have insight into their actions and those of staff using cameras. Others viewed violence and aggression as just “part of the job”. Some staff even felt that BWCs could instigate incidents and increase patient distress and aggression. BWCs were viewed by some as a punitive measure, contributing to patients’ feelings of criminalization and intimidation. Some staff felt that BWCs could negatively impact therapeutic relationships and contribute to a “them and us” culture.

Staff had concerns about the impact of BWC use on patients’ privacy and dignity, with some staff feeling there needed to be limits around their use (e.g. a rule to not use them in private spaces such as patient bedrooms and bathrooms). However, it was acknowledged that these boundaries could be difficult to adhere to in practice. Staff emphasised the need for flexibility and to be able to decide not to use cameras in situations where they could cause harm, enabling a person-centred approach.

Whilst flexibility was felt to be important, staff also identified inconsistency in BWC implementation across trusts as an issue, with some feeling that national guidelines are needed. Staff said that guidelines and policies need to be developed considering how, why, what, where and when to use BWCs, with leadership and management involvement, and consultation with patients and staff, from the beginning of the implementation process. Feedback from staff and patients was viewed as important for ensuring continued improvement of BWC implementation. They noted that policies should include robust governance and data management plans, supported by good IT infrastructure and support, to help assure patients and carers of the safety of their data.

Staff also raised ethical and legal concerns around patient consent and the potential for BWCs to be used as a substitute for good care and safe staffing. They felt it was important to inform patients about how, why and when BWCs are used, and their rights in relation to them, to ensure transparency and hold staff to account to use them correctly. Staff recognised the importance of providing this information continually, since patients’ health and capacity can fluctuate during their inpatient stay.

Some staff found BWCs small, easy to use, user-friendly and viewed them as having little direct impact on their daily routines. However, others expressed concerns about useability, especially for staff less confident with technology. Practical challenges included overheating causing skin discomfort, cameras pulling on clothing, infection control concerns, charging and storage issues and the potential for BWCs to be lost or damaged. There were issues with staff forgetting to wear, activate or recharge cameras. The dynamic and reactive ward environment means that staff often react to incidents quickly, which can result in them forgetting or failing to use BWCs. There were mixed views on whether reviewing BWC footage saved time or added to workloads, with varying procedures across services for footage management.

There was a lack of consistency in staff understanding of BWC uses and procedures, and in BWC training received, with some staff receiving no or very limited training. BWC training was widely thought to be insufficient, with staff expressing a need for more comprehensive training, delivered on a rolling basis. They felt this should cover not just operational aspects, but, for example, ethical considerations, decision-making around BWC use, communication techniques, use in sensitive situations and how BWC use could impact on therapeutic relationships, treatment and recovery.

Some staff saw BWCs as top-down directives from senior management and policy stakeholders with limited frontline experience, contributing to poor staff buy-in and concerns about misspent funds. Some staff felt that de-escalation skills, communication and positive relationships need to be the primary tools for violence reduction, before BWCs, and that there needs to be greater investment in staff training and support, rather than in new technologies [51, 52, 55, 68].

#### Patients

Some patients reported feeling safer with BWCs due to them providing evidence to support their claims and protect them against staff misconduct. However, others felt BWCs did not improve safety and considered the footage biased since the cameras are controlled by staff and only capture incidents when staff are present. Some patients expressed concerns that BWCs negatively impacted their recovery, privacy and dignity, and could act as a “trigger”, particularly for people experiencing paranoia, psychosis or who had previous negative experiences of being filmed. Many patients felt less comfortable being filmed in private spaces, such as bedrooms and bathrooms, compared to communal ward spaces, and so felt that BWC use in these areas should be avoided unless “really necessary”.

Similar to staff, some patients felt that BWCs fail to address the systemic causes of violence and aggression, and that any improvements in safety are due to increased staff awareness and reflexivity, rather than changes in patient behaviour. Some patients viewed BWCs as punitive, contributing to feelings of criminalization and exacerbating power imbalances between patients and staff. Patients also raised ethical and legal concerns about the role of patient consent in BWC use, and reported receiving a lack of information about how and why the cameras were used and their rights.

Patients noted inconsistency in how BWCs are implemented and identified a need for policies and procedures to be developed around their use, including clear boundaries, to ensure a consistent culture and understanding. Some called for a national directive around BWC use to ensure greater consistency across services. They emphasised the importance of consulting with patients throughout the implementation process, to ensure that policies and procedures are sensitive to their needs and concerns. People who are autistic, experiencing psychosis or who have a history of trauma, were identified as particularly important to involve given the potential negative impact of BWCs on these groups [51, 52, 55, 68].

### GPS electronic monitoring and wearable sensors

None of the included studies explored staff, patient or family/carer post-implementation experiences of GPS electronic monitoring or wearable sensors.

### Research objective 3: what is the effect, including unintended consequences, harms and benefits, of surveillance-based technologies in inpatient mental health settings for outcomes such as patient and staff safety and patient clinical improvement?

Fifteen studies reported outcomes on the effectiveness of surveillance strategies in inpatient mental health settings [40–47, 52, 55, 56, 61, 65–67]. Overall, the findings were limited and mixed. The findings below are reported by type of surveillance and tabulated in Table 10.

**Table 10:** Quantitative evidence for the impact of surveillance technologies in inpatient mental health settings

Author and year	Study design	Setting	Intervention and control group	Analysis method	Results	Conflicts of interest and MMAT rating
**Vision-Based Patient Monitoring and Management (VBPMM)**						
Barrera et al. [47]	Service improvement project/ feasibility study (Pre-post design with concurrent control period)	An adult acute male inpatient mental health ward	Intervention: VBPMM-assisted observations Control/comparison: In the initial implementation phase, the VBPMM-assisted observations ran in parallel to the existing observations protocol.	Pearson’s correlations between measures at T1 (on admission to a bedroom with sensors) and T2 (the point of moving to a bedroom without sensors). And comparison of VBPMM-assisted observations and standard observations in the early implementation phase.	Insomnia: Insomnia Severity Index scores significantly decreased the longer patients slept in a bedroom with VBPMM (Pearson correlation: 0.403; two-tailed *p* = 0.016; *n* = 35). Length of stay: Significant positive correlation between nights in rooms with VBPMM and length of stay (Pearson’s correlation: 0.410; two-tailed *p* = 0.003; *n* = 50). However, the duration of their hospital admission (*n* = 47, mean = 44.01, SD 43.62) was not significantly longer than the duration of admission of all patients admitted to the ward in the 12 months prior to VBPMM being used (*n* = 131; mean = 40.40; SD 35.90) (*T* = − 0.437, df = 65.56, two-tailed *p* = 0.664). Medication use: No significant difference in the frequency of hypnotic and anxiolytic medication use (including zopiclone, promethazine and benzodiazepines) between T1 and T2 (*p* value not reported). Rapid tranquillisations: No significant difference in the frequency of rapid tranquillisation between T1 and T2 (*p* value not reported). Care quality: 100% match of vital sign reports between observations with and without VBPMM. Complaints and damage: Ward incident reports showed no incidents or negative comments were reported related to VBPMM.	Conflicts of interest: Yes MMAT rating: Low
Buckley et al. [41]	Economic analysis study utilising a cost-calculator approach (using data from five observational before and after studies)	Seven adult acute inpatient mental health wards, across four NHS trusts in England, and seven older adult inpatient mental health wards across three NHS trusts in England. One trust was Coventry and Warwickshire Partnership NHS Trust. Other trusts chose to remain anonymous.	Intervention group: Wards with VBPMM implemented Control/comparison: No control group. Comparison group was inpatient mental health care without VBPMM.	Updated a previous economic model with additional cost and clinical data for analysis [42, 43]. A 12-month time horizon was used. Data used to populate the model were informed by sources including five before-and-after studies conducted across five NHS mental health trusts. The outcomes from the clinical studies were standardised to “per occupied bed days”. The data were scaled to an average ward with 16 beds and 90% occupancy. Where clinical data from multiple trusts was used, weighted averages were calculated based on the number of bed days during the study period. Primary outcomes from the economic model include cost per occupied bed day, cost per patient, cost per average ward per year, and cost to mental health NHS trusts. The economic model also considers whether cost savings are cash releasing (produce a monetary return) or opportunity cost saving (resulting from resources being released which could be used for other activities). The model considers one-to-one observations to be the only event with a cash-releasing cost saving component, as it can require additional resource from a ward beyond planned staffing levels and so bank and agency staff are used. Return on investment (ROI) was also calculated.	Cost-effectiveness: **Adult acute inpatient mental health wards** *Estimated effect of adopting VBPMM on adult acute mental health inpatient wards (number without VBPMM, number with VBPMM, percentage reduction with VBPMM):* • Night-time observations (seconds per observation): 25.8, 14.3, 44.7% • One-to-one observations (hours per occupied bed day)—cash releasing: 1.63, 1.20, 26.2% • One-to-one observations (hours per occupied bed day)—opportunity cost saving: 0.68, 0.55, 20.4% • One-to-one observations (hours per occupied bed day)—total: 2.38, 1.79, 24.4% • Self-harm incidents (number per occupied bed day): 0.0094, 0.0052, 44% *Estimated cost savings:* Estimated that when VBPMM is implemented on an average-sized acute adult mental health inpatient ward in England with 90% occupancy: Savings per adult acute ward per year: • £93,433 (NHS & PSS perspective). • £89,305 (NHS mental health trust perspective). Incremental benefits for adult acute wards (cash releasing only): • £46,437 (both NHS & PSS perspective and NHS mental health trust perspective). Return on investment in relation to total benefits on adult acute wards: • 3.17 (NHS & PSS perspective) • 3.03 (NHS mental health trust perspective) Return on investment in relation to cash releasing benefits only on adult acute wards: • 1.58 (both NHS & PSS perspective and NHS mental health trust perspective). If VBPMM was implemented across all of NHS England acute mental health services (18,400 beds for mental healthcare available, of which 65% are acute services): • Estimated £69 million in cost savings, assuming 90% average occupancy. • Estimated that around half of these cost savings would be cash-releasing to the healthcare system. These acute adult inpatient service estimates were based on the following calculations (NHS & PSS perspective, NHS mental health trust perspective): • Total costs of a VBPMM system: £29,457, £29,457 • Reduction in cost of night-time observations: £7380, £7380 • Reduction in cost of one-to-one observations—cash releasing: £75,895, £75,895 • Reduction in cost of one-to-one observations—opportunity cost saving: £27,038, £27,038 • Reduction in cost of self-harm incidents: £12,578, £8449 • Total cost without VBPMM: £467,487, £458,104 • Total benefits with VBPMM (cost saving excluding cost of VBPMM): £122,891, £118,762 **Cost effectiveness:** Older adult inpatient mental health services *Estimated effect of adopting VBPMM on older adult acute inpatient mental health wards in England (number without VBPMM, number with VBPMM, percentage reduction with VBPMM):* • Night-time observations (seconds per observation): 20.25, 10.12, 50% • One-to-one observations (hours per occupied bed day)—cash releasing: 1.49, 0.88, 40.4% • One-to-one observations (hours per occupied bed day)—opportunity cost saving: 1.11, 0.32, 70.9% • One-to-one observations (hours per occupied bed day)—total: 2.61, 1.21, 53.4% • Bedroom falls at night (number per occupied bed day): 0.014, 0.0071, 48% • A&E visits resulting from bedroom falls at night (number per occupied bed day): 0.0019, 0.0006, 68% • Emergency services callouts resulting from bedroom falls at night (per year): 0.005, 0.002, 49% *Estimated cost savings:* Estimated that when VBPMM is implemented on an average-sized older adult mental health inpatient ward in England with 90% occupancy: Savings per older adult inpatient mental health ward per year: • £413,651 (NHS & PSS perspective) • £265,376 (NHS mental health trust perspective) Incremental benefits (cash releasing only) for older adult inpatient wards: • £77,628 (both NHS & PSS perspective and NHS mental health trust perspective) Return on investment in relation to total benefits for older adult inpatient wards: • 14.04 (NHS & PSS perspective) • 9 (NHS mental health trust perspective) Return on investment in relation to cash releasing benefits only for older adult inpatient wards: • 2.63 (both NHS & PSS perspective and NHS mental health trust perspective) If VBPMM was implemented across all of NHS England for older adult mental health services, with around 18% beds for mental healthcare assigned to older adults: • It would lead to estimated cost savings of approximately £89 million. • Around one-third of these cost savings would be cash-releasing to the healthcare system. These older adult inpatient service calculations are based on the following calculations (NHS & PSS perspective, NHS mental health trust perspective): • Total costs of a VBPMM system: £29,472, £29,472 • Reduction in cost of night-time observations: £29,969, £29,969 • Reduction in cost of one-to-one observations—cash releasing: £107,101, £107,101 • Reduction in cost of one-to-one observations—opportunity cost saving: £140,335, £140,335 • Reduction in cost of bedroom falls at night: £142,948, £17,443 • Reduction in cost of A&E visits resulting from bedroom falls at night: £18,308, £0 • Reduction in cost of emergency services visits resulting from bedroom falls at night: £4461, N/A • Total costs without VBPMM: £854,767, £558,989 • Total benefits with VBPMM (cost saving excluding cost of VBPMM): £443,123, £294,848 The largest driver of cost savings on both adult acute inpatient wards and older adult inpatient wards was the reduction in one-to-one observation hours.	Conflicts of interest: Yes MMAT rating: Low
Clark et al. [40]	Proof of concept quality improvement project (uncontrolled pre-post design)	A women’s PICU in a hospital in South London. Age of the inpatient population not specified.	Intervention: VBPMM in seclusion Control/comparison: No control group. Comparison was baseline data for the three months prior to VBPMM installation	Mann–Whitney U and binomial tests were used to make pre-post VBPMM comparisons	Restraint and restrictive practices: VBPMM use did not significantly change seclusion session duration (*p* = 0.61; Mann–Whitney *U* test) or seclusion frequency (*p* = 0.49; binomial test).	Conflicts of interest: Yes MMAT rating: Low
Malcolm et al. [42]	Economic analysis study utilising a cost-calculator approach (using data from a single centre observational before and after study)	An adult PICU	Intervention: 12-month period where VBPMM was implemented in a PICU Control/comparison: No control group. Comparison was the 12-month period before VBPMM was implemented in the PICU	This cost-calculator approach to economic analysis focused on comparing the number of clinical events, observations and associated costs following the introduction of VBPMM compared to standard care alone. A 12-month time horizon was used. Quality of life was not captured in the model. Scenario analysis was conducted to test the uncertainty of results using statistical significance of key inputs.	Costs: VBPMM + standard care was estimated to reduce costs by £72,286 per averaged sized ward per year, equivalent to £880 per patient per year, or £18 per occupied bed day, leading to an estimated cost saving to the NHS per year of £5,541,294 if rolled-out to all PICUs in England. *Breakdown of the costs calculated:* (*Standard care, VBPMM* + *standard care, Difference)* • Cost of night-time observational hours: £268, £158, − **£109** • Cost of one-to-one observation hours: £10,749, £9943, − **£806** • Cost of assaults: £227, £167, − **£60** • Cost of rapid tranquillisation event: £562, £338, − **£223** • Cost of VBPMM: £0, £319, £319 • Total cost per patient: £11,806, £10,926, − **£880** • Total cost per occupied bed day: £246, £228, − **£18** • Total cost per average sized ward per year: £970,193, £897,907, − **£72,286** • Total cost to the NHS per year: £74,381,491, £68,839,567, − **£5,541,924** **Scenario analysis only including significant events (night-time observational hours and rapid tranquillisation events, not one-to-one observation hours or assaults) (Standard care, VBPMM + standard care, Difference):** • Total cost per occupied bed day: £246, £246, **£0** • Total cost per patient: £11,806, £11,792, − **£14** • Total cost per average sized ward per year: £970,193, £969,053, ** − £1140** • Total cost to the NHS per year: £74,381,491, £74,294,073, ** − £87,418** The authors state that removing events that were not statistically significant from the analysis still resulted in a marginal cost saving with the introduction of VBPMM. **Scenario analysis investigating potential effect that including VBPMM can have just relating to one-to-one observation hours:** “A 4% reduction in one-to-one hours is expected to be needed to break even in-year if considering only the cash releasing aspect of one-to-one observation hours (with all other inputs as per the base case scenario), compared to 7% as estimated in the base case. If considering all potential cost savings, including opportunity costs, it is estimated that cost savings from a reduction in other events would be sufficient to break even without any reduction in one to one observation hours”. **Deterministic sensitivity analysis** showed “the key independent drivers of the results are the reduction in one to one observation hours, the number of baseline one to one observation hours and the cost of one to one observation time per hour. Only the reduction of one to one observation hours with the VBPMM changes the direction of the results of the analysis to cost incurring. This is likely due to the wide confidence interval around this parameter”.	Conflicts of interest: Yes MMAT rating: Low
Malcolm et al. [43]	Economic analysis study utilising a cost-calculator approach (using data from two single centre observational before and after studies, one published and one unpublished)	Two acute adult mental health wards (within our review’s scope) and two older adult mental health wards for dementia (outside of our review’s scope)	Intervention: 12-month period where VBPMM was implemented in two acute adult mental health wards Control/comparison: No control group. Comparison was a 12-month period before VBPMM was implemented in the adult acute mental health wards	This cost-calculator approach was used to compare the introduction of VBPMM as an adjunct to standard care versus standard care alone. The model compared data pre- and post-VBPMM implementation using a 12-month baseline period. The model estimated cost per occupied bed day, cost per patient, annual cost per average-sized ward, and total cost to NHS mental health trusts across England. Costs were modelled from an NHS perspective over a 12-month time horizon. Scenario analysis was conducted to test the uncertainty of results using statistical significance of key inputs.	Costs: VBPMM + standard care was estimated to result in cost savings of £29,827 per average sized ward per year, or £272 per patient, or £6 per occupied bed day, leading to an overall estimated cost saving to the NHS per year of £22,295,434 if rolled-out to all acute adult mental health inpatient wards in England. *Summary of the costs calculated:* *Standard care cost, VBPMM* + *standard care cost, Incremental)* • Cost of observations (£ per patient): £107, £63, − **£44** • Cost of one-to-one observations (£ per patient): £1813, £1444, − **£369** • Cost of self-harm incidents (£ per patient): £231, £129, − **£101** • Cost of VBPMM (£ per patient): £0, £242, **£242** • Total cost per patient (£): £2151, £1879, − **£272** • Total cost per occupied bed day (£): £45, £39, − **£6** • Total cost per average sized ward per year (£): £235,676, £205,849, ** − £29,827** • Total cost to the NHS per year (£): £176,167,573, £153,872,139, ** − £22,295,434** The main driver of cost savings was the reduction in one-to-one observations. Threshold analysis showed a 33% reduction in one-to-one observations is needed in an average sized adult acute mental health ward to generate cash releasing savings. When all potential cost savings were considered (including opportunity costs), a 7% reduction in one-to-one observations was estimated to be sufficient to break even. Scenario analyses showed that excluding one-to-one observations from the model resulted in VBPMM no longer providing cost savings: **Scenario analysis excluding one-to-one observations results (Standard care, VBPMM + standard care, difference):** Total cost per occupied bed day: £45, £47, **£2** Total cost per patient: £2151, £2248, **£97** Total cost per average sized ward per year: £235,676, £246,309, **£10,633** Total cost to the NHS per year: £176,167,573, £184,115,799, **£7,948,226** **Deterministic sensitivity analysis** showed that the key drivers of the results are reduced one-to-one observation hours, reduced self-harm incidents per year, one-to-one weighted staff cost for observation time per hour, number of one-to-one observation hours per occupied bed day, and average length of stay per patient.	Conflicts of interest: Yes MMAT rating: Low
Ndebele et al. [46]	Single centre pragmatic study	A male PICU ward at Coventry and Warwickshire NHS Partnership Trust. Age of patients not specified.	Intervention group: Data collected 12-month post-VBPMM implementation on the PICU ward Control/comparison: No control group. Compared incident rates to the 12-month period pre-VBPMM on the same PICU ward	Changes in numbers of assaults and the use of rapid tranquillisation pre- and post-use of the system were assessed. A bootstrap method with resampling was applied over subjects to assess the level of significance, due to the small sample size. Data was normalised for bed occupancy for the study duration.	Assaults • There was a significant 37% decrease in assaults overall (*p* = 0.0036) after implementing VBPMM. • There was a non-significant 26% decrease in the number of assaults in bedrooms after implementing VBPMM (*p* = 0.33). • There was a small increase in patient-to-patient assaults specifically in bedrooms after implementing VBPMM (significance testing not reported). • Stated that the severity of assault remained broadly unchanged, suggesting that VBPMM may assist staff in managing all types of incident equally (though no description of how severity was assessed). * Summary of assaults (baseline period, active period, % change, % adjusted to occupied bed days)* • All assaults: 244, 145, − 41, − 37 • Patient-to-patient assaults: 52, 36, − 31, − 26 • Patient-to-staff assaults: 192, 109, − 43, − 39 • Bedroom assaults: 42, 29, − 31, − 26 • Bedroom patient-to-patient assaults: 2, 4, 100, 113 • Bedroom patient-to-staff assaults: 40, 25, − 38, − 33 • Occupied bed days: 3704, 3473 Rapid tranquillisations: • There was a significant 40% decrease in rapid tranquillisation events related to assaults (from 85 in the baseline period to 48 in the active period) after implementing VBPMM (*p* = 0.0018).	Conflicts of interest: Yes MMAT rating: Low
Ndebele et al. [45]	Mixed methods non-randomised controlled pre-post evaluation within a pilot study	At Caludon Centre, Coventry & Warwickshire Partnership NHS Trust (CWPT), a purpose-built facility, based on the University Hospital Coventry and Warwickshire (UHCW) site, providing inpatient and outpatient adult mental health care	Intervention group: two acute wards fitted with VBPMM (22-bed female and 20-bed male) Control/comparison: Control group was two acute wards without VBPMM selected based on the similarity of the patient cohort, ward size and clinical ways of working	Rates of self-harm and ligatures were analysed for both the observational and control wards before (baseline period) and after (active period) the VBPMM was implemented on the intervention wards. Confounder analysis was conducted via interviews with ward managers. The ward percentage change in incident rates between the baseline and active periods was calculated for the intervention and control wards. A relative percentage change in incident rates was calculated between the ward percentage change for the intervention wards and control wards. Incident data were normalised for ward monthly occupancy. Statistical significance was evaluated using the basic bootstrap method (aka “Reverse Percentile Interval”) with resampling applied over patients. Incident rates were calculated to assess change in self-harm and ligature incidents across the two groups.	Self-harm incidents: There was a significant relative percentage change of − 44% (*p* < 0.002, 95% CI to [− 100%, − 14%]) in the number of self-harm incidents in the bedroom, which includes en suite bathrooms, in the active period on the intervention wards compared to the control wards. There was a non-significant ward percentage change in incidents of self-harm in bedrooms in the active period compared to the baseline period on the intervention wards (− 22% (*p* = 0.32, 95% CI [− 100, + 19%]). Ligature incidents: There was a significant relative percentage change of incidents of ligatures in the bedroom in the active period on the intervention wards compared to the control wards (− 48% (*p* < 0.001, 95% CI [–100%, − 16%])).There was a -68% (*p* < 0.001, 95% CI [− 100%, − 40%]) relative percentage change in en suite bathroom ligatures in the active period across the intervention wards.	Conflicts of interest: Yes MMAT rating: Low
**Closed circuit television (CCTV)/video surveillance**						
Simpson et al. [61]	Cross-sectional survey study	136 acute adult psychiatric wards across London, Central England and North England	No intervention or control groups—was a cross-sectional survey of psychiatric wards	Spearman’s *r* correlations	Alcohol use on ward: No significant association between CCTV for viewing those leaving the ward and alcohol use on the ward (Spearman’s *r* = − 0.083; *p* = 0.345). Other substance use on ward: No significant association between CCTV for viewing those leaving the ward and other substance use on the ward (Spearman’s *r* = − 0.059; *p* = 0.497).	Conflicts of interest: No MMAT rating: High
Vartiainen & Hakola [66]	Pre-post study	Four closed adult male wards in the Niuvanniemi hospital in Finland.	Intervention: Wards 3 and 4 were renovated, including adding CCTV and reducing the number of beds. Control/comparison: Control groups were wards 1 and 2 which were also renovated, with the number of beds reduced, but no CCTV added.	Mann Whitney U tests were used to compare patient and staff ratings of ward atmosphere, subjective mental health and paranoid traits on each of the wards before the renovations and after. No significance testing of changes in violent acts was conducted. There were no statistical comparisons of changes in outcomes on intervention and control wards.	Violence and aggression: Violent acts reduced from a total of 70 on wards 3 and 4 in the year before implementing CCTV, to 57 during the year following introducing CCTV. Significance testing was not reported. Ward atmosphere: • There was a significant improvement in staff ratings of ward atmosphere on ward 4 (a CCTV monitored ward) (*p* < 0.01) but not on any of the other wards. • There were no significant changes in patients’ ratings of ward atmosphere on any of the wards (*p* > 0.05). Mental health: • There were no significant changes in staff or patients’ ratings of subjective mental health or paranoid traits on any of the wards (*p* > 0.05). Complaints and damage: During two years of TV monitoring, no cameras were damaged.	Conflicts of interest: No MMAT rating: Low
Warr et al. [67]	Mixed methods study (qualitative interviews and cross-sectional quantitative component)	Montpellier adult low-secure unit in England	Intervention: CCTV used to monitor consenting patients in their bedrooms at night Control/comparison: None	Compared the frequency and nature of “untoward incidents” during the day (CCTV not in operation) and at night (CCTV in operation) during a 12-month period. It is unclear whether any statistical significance testing was conducted.	Violence and aggression: 45 incidents (all verbal or physical abuse to staff or other patients) reported during the 12-month period, 8 of these were at night. • There were therefore fewer incidents at night (when CCTV was active) than during the day (when it was not) but the authors reported that this is likely due to the fact that most patients were asleep at night. • The nature of the incidents did not differ significantly from those during the day. There was nothing in the reports to suggest an association with the presence or use of CCTV, or the choice of the patients to be observed via CCTV or not.	Conflicts of interest: No MMAT rating: Medium
**Body-worn cameras (BWCs)**						
Ellis et al. [44]	A quasi-experimental repeated measures design	Seven West London Trust mental health adult wards, including: two wards for local services admissions (male and female), a PICU (male), a low secure forensic ward (male), medium secure ward (female) and two enhanced medium secure wards (both female).	Intervention: BWCs were introduced on a rolling basis, ward-by-ward. Control/comparison: No control group. Comparisons were made pre- and post-implementation of BWCs using distinct 4-month periods that were matched depending on the date of introduction of BWCs to the ward.	The seven wards were grouped into three categories (1 & 2—local services admissions; 3 & 4—PICU and low-secure forensic ward; 5, 6 & 7—medium and enhanced medium units). Incidents were categorised into four levels of seriousness, from least to most: 1—verbal aggression, 2—violence not requiring restraint, 3—restraint not including when tranquillising injection was required, 4—restraint resulting in tranquillising injection. *T* tests were used to analyse patterns of change across the three groupings and across the four ward types.	Incidents (ranging from verbal aggression to violence without restraint, violence with restraint, and restraint resulting in rapid tranquillisation): • Found no significant changes in any level of incident overall. • There was a significant reduction in the seriousness of incidents between the before period (M = 2.4, SD = 0.918) and after period (M = 2.04, SD = 0.083) on wards 1 & 2; *t*(115.994) = 2.459, *p* = 0.015. No significant changes in the seriousness of incidents on the other five wards (*p* values not reported).	Conflicts of interest: Yes MMAT rating: Low
Foye et al. [52]	Mixed methods study with repeated measures design	An adult acute inpatient ward and an adult psychiatric intensive care unit in a London mental health trust.	No control group. Compared the six-month period pre-BWC implementation, the six-month pilot period when BWCs were used, and the 6-month post-pilot period when BWCs had been removed.	Incident reports from Datix were binary coded into aggregate variables to examine violence and aggression, self-harm, and other conflict. Multivariate analyses of variance (MANOVA) were used to identify differences in type of incident (violence against person, violence against object, verbal aggression, self-harm, conflict) for each ward. MANOVA was also used to examine differences in incident outcomes (severity, police involvement, use of restrictive practice) across pre-trial, trial, and post-trial periods for each ward. Linear regressions assessed the relationship between BWC use and incident outcome (severity, use of restrictive practice, police involvement).	Incident type: *Acute ward* • On the acute ward, there was no significant effect of trial period on incident type (F(10, 592) = 1.703, *p* = .077, Wilk’s Λ = 0.945). There was therefore no significant difference in the type of incidents occurring before, during, and after the BWC pilot phase. *PICU:* • On the PICU, there was a significant effect of trial period on incident type (F(10, 490) = 4.252, *p* < .001, Wilk’s Λ = 0.847). • Verbal aggression was significantly higher in the post-trial period compared to the pre- and trial periods (*p* < 0.001). • There were no significant differences in violence against a person (*p* = 0.162), violence against an object, or conflict behaviour. Incident outcomes *Acute ward* • On the acute ward, there was a significant effect of trial period on incident outcomes (F(6, 596) = 10.900, *p* < .001, Wilk’s Λ = 0.812). • Incident severity was significantly higher in the trial (*p* = 0.05) and post-trial periods (*p* = 0.05) compared to the pre-trial period. • Use of restrictive practice and police involvement were significantly lower in the post-trial period compared to the pre-trial and trial periods (all *p* < 0.001). *PICU* • On the PICU, there was a significant difference in incident outcomes across the trial periods (*F*(6, 494) = 12.907, *p* < .001, Wilk’s Λ = 0.747). • There was no significant difference in incident severity or police involvement. • However, use of restrictive practice was significantly higher in the pre-trial period (*p* < 0.001), reducing in the test period (*p* < 0.001), and reducing further in the post-trial period (*p* < 0.001).	Conflicts of interest: None MMAT rating: Low
Hardy et al. [55]	Mixed methods pre-post pilot study	Berrywood Hospital, an adult psychiatric facility in Northampton, England, run by Northamptonshire Healthcare NHS Foundation Trust. The five wards in the pilot included one male and one female recovery, one low secure unit, one acute.	Intervention: BWCs were introduced to the hospital Control/comparison: No control group. Routinely collected data during the period of this study was compared with routinely collected data for the same time period before the intervention.	Descriptive analysis to compare patient outcomes before and after the intervention. No significance testing.	Violence and aggression: • Verbal abuse increased on 3/5 wards, decreased on 1/5 wards and stayed the same on 1/5 wards. • Violence reduced on 3/5 wards and increased on 2/5. Restraint: • Low level restraint increased on 2/5 wards, reduced on 2/5 wards and stayed the same on 1/5. • Emergency restraint reduced on 3/5 wards and increased on 2/5 wards. Complaints and damage: • Three complaints were made during the study period, one of which was withdrawn. None were related to a specific incident or restraint. • During the comparison period pre-BWC implementation, there were three complaints made by patients, and one withdrew. One patient made six complaints and one made two, both complained about an instance of restraint. • No damage to cameras was reported.	Conflicts of interest: No MMAT rating: Low
**Global Positioning System (GPS) electronic monitoring**						
Murphy et al. [56]	Pre-post study	River House, an adult medium-secure unit in South London and Maudsley NHS Foundation Trust (107 male beds and 15 female beds)	Intervention: Episodes of leave using GPS electronic monitoring during a 3-month period (1st January 2011–31st March 2011). Control/comparison: No control group. Comparison was episodes of leave during a corresponding 3-month baseline period the previous year (1st January 2010–31st March 2010) prior to the introduction of GPS electronic monitoring	The average total cost per patient was calculated for the intervention and comparison period and included leave violations, staff costs and electronic monitoring overheads. Chi-squared tests were used to determine whether the 2010 and 2011 groups were matched for demographic details including age, sex and diagnosis. As some patients appeared in both cohorts, costs between the 2010 and 2011 groups were compared using a regression model clustering on the patient ID number.	Leave violations: There were six leave violation incidents in the 2010 and 2011 groups. In 2010, two patients absconded from escorted leave and four failed to return from unescorted leave. In 2011, six patients failed to return on time and there were no episodes of absconding. Cost-effectiveness: • Total **staff costs** in the 2010 group (without electronic monitoring): £163,390 • Total **staff costs** in the 2011 group (with electronic monitoring): £161,050 • Lower staff costs in the 2011 group, despite an overall greater number of leave episodes, indicates a higher proportion of unescorted leave. • Additional **electronic monitoring costs** for the 2011 group: £34,653 • Total expenditure in the 2011 group: £195,730 • Average total cost was £1702 per patient in the 2010 group (without electronic monitoring) and £1617 per patient in the 2011 group (with electronic monitoring). • Total costs per patient before and after introduction of electronic monitoring were *not* significantly different.	Conflicts of interest: No MMAT rating: Medium
Tully et al. [65]	Pre-post study	The South London and Maudsley medium secure service in England (comprising two medium secure units in South London at the time of the study). Age of the inpatient population not specified.	Intervention: Episodes of leave during a 4-month follow-up period 1-year post-implementation of electronic monitoring (1st Dec 2010–31st Mar 2011) and 2-years post-implementation (1st Dec 2011–31st Mar 2012). Control/comparison: No control group. Comparison was episodes of leave during a corresponding 4-month baseline period before electronic monitoring was introduced (1st Dec 2009–31st Mar 2010).	Chi-squared tests were used to analyse the association between leave type (escorted/unescorted) and period studied (2009/10 [pre-implementation], 2010/11, and 2011/12 [post-implementation]). Logistic regression analyses were used to determine the effect of year on leave violation (no incident vs leave violation). The variable “period” was coded into two dummy variables (each of the two follow-up periods), with “baseline” period as the reference category.	Type of leave episodes: • There was a significant association between type of leave episode and year (χ^2^ (df,3) = 1.008.5, *p* < 0.001), where leave episodes after the introduction of electronic monitoring were more likely to be unescorted. Leave violations: • Leave episodes in the second follow-up period were significantly less likely to lead to an incident of leave violation (OR = 0.21, CI 0.06–0.77), but not in the first follow-up (OR = 0.42, 95% CI 0.15–1.19). Complaints: The electronic monitoring system was challenged on two occasions by patients—reasons for this were not provided.	Conflicts of interest: No MMAT rating: Low

Acronyms: *BWCs* body-worn cameras, *CCTV* closed circuit television, *CI* confidence interval, *GPS* Global Positioning System, *MMAT* Mixed Methods Appraisal Tool, *NHS* National Health Service, *PICU* psychiatric intensive care unit, *PSS* Personal Social Services, *ROI* return on investment, *SD* standard deviation

#### Vision-Based Patient Monitoring and Management (VBPMM)

Seven studies reported on the effect of VBPMM [40–43, 45–47]. All studies reported on Oxevision by Oxehealth. All studies were rated low quality, and all declared conflicts of interest [40–43, 45–47]. Study designs included a mixed methods non-randomised controlled pre-post evaluation within a pilot study, which compared two intervention wards with VBPMM to two control wards without VBPMM [45], three economic analysis studies utilising cost-calculator approaches [41–43], two uncontrolled pre-post designs [40, 46] and a pre-post study with a concurrent control period [47].

##### Self-harm and ligature incidents

One study investigated VBPMM’s effect on self-harm and ligature incidents; it reported a significant relative reduction in self-harm and ligature incidents in bedrooms on the VBPMM wards *compared* to the control wards. However, when considering the VBPMM wards *alone*, there was a significant decrease in ligature incident rates, but not in self-harm rates, after introducing VBPMM [45].

##### Assaults

One study evaluated the impact of VBPMM on assaults; it reported a significant overall reduction in assaults on a PICU ward following its implementation. However, there was no significant change when specifically considering assaults in patient bedrooms. An increase in patient-to-patient assaults in bedrooms was found, but the statistical significance of this change was not reported [46].

##### Restrictive practices

Three studies reported on VBPMM’s effect on restrictive practices [40, 46, 47]. Barrera et al. [47] reported no significant effect on rapid tranquillisation frequency, whilst Ndebele et al. [46] reported a significant decrease in rapid tranquillisation events related to assaults after introducing VBPMM. Clark et al. [40] reported no significant impact upon seclusion session frequency or duration.

##### Clinical outcomes

One study investigated VBPMM’s effect on clinical outcomes [47]. It reported that insomnia severity significantly decreased the longer patients slept in a bedroom with VBPMM. There was a significant positive correlation between nights in rooms with VBPMM and hospital length of stay, although there was no significant difference in patients’ average hospital admission duration post-VBPMM and the average admission duration for all patients admitted to the ward in the 12 months before VBPMM introduction. There was also no significant difference in the use of hypnotic and anxiolytic medication [47].

##### Care quality

One study reported VBPMM’s effect on care quality-related outcomes [47]. It reported a 100% match of vital sign reports between observations with and without sensors.

##### Cost-effectiveness

Three studies investigated the cost-effectiveness of VBPMM [41–43], all utilising cost calculator approaches. Malcolm et al. estimated that VBPMM in addition to standard care, compared to standard care alone, reduced costs on a PICU by £72,286 per ward per year, equivalent to £880 per patient per year, or £18 per occupied bed day [42]. They estimated that if rolled-out to all adult PICUs in England, VBPMM would lead to an estimated cost saving to the NHS per year of £5,541,924. These calculations were based on estimates that VBPMM would cost £319 per patient (including annual licence fees, installation and cabling and staff training costs) and that it would reduce costs per patient by £109 for night-time observation hours, £806 for one-to-one observation hours, £60 for assaults and £223 for rapid tranquillisation events. Full breakdowns of these costs and calculations are available in Table 10. The key driver of these savings was 36 h of staff time saved per patient per year, primarily driven by a decrease in one-to-one observation hours. Scenario analyses showed that these results were robust to statistically significant changes in input parameters [42].

Malcolm et al. [43] estimated that VBPMM in addition to standard care, compared to standard care alone, reduces costs on acute adult mental health wards by £29,827 per average sized ward per year, equivalent to £272 per patient per year, or £6 per occupied bed day. They estimated that if rolled-out to all acute adult mental health wards in England, VBPMM would lead to an estimated cost saving to the NHS per year of £22,295,434. This was based this on estimates that VBPMM would cost £242 per patient (including licence fees, annualised installation and cabling and staff training costs) and that it would reduce costs per patient by £44 for night-time observations, £369 for one-to-one observations and £101 for self-harm incidents. The main driver of cost savings was the reduction in one-to-one observations. They reported that threshold analysis showed a 33% reduction in one-to-one observations is needed in an average-sized adult acute mental health ward to generate cash savings. Scenario analyses showed that if one-to-one observations were removed from the economic model, VBPMM no longer provided cash savings [43]. Full breakdowns of these costs and calculations are available in Table 10.

Buckley et al. [41] calculated the cost impact of implementing VBPMM on an average-sized acute adult inpatient mental health ward, and an average-sized older adult inpatient mental health ward, in England, from the perspective of NHS and Personal Social Services (PSS) and from the perspective of an NHS mental health Trust. They calculated that per average-sized adult acute inpatient mental health ward, per year, VBPMM would result in cost savings of £93,433 (NHS & PSS) or £89,305 (NHS mental health trust). This equates to a Return on Investment (ROI) of 3.17 (NHS & PSS) or 3.03 (NHS mental health trust). These calculations were based on estimates that a VBPMM system would cost £29,457 per ward per year, and that introducing VBPMM would reduce costs per ward per year by £7380 for night-time observations, £75,895 for one-to-one observations (cash-releasing), £27,038 for one-to-one observations (opportunity cost saving) and £12,578 (NHS & PSS) or £8449 (NHS mental health trust) for self-harm incidents. They identified a reduction in one-to-one observations as the main driver of these cost savings. They predicted that if VBPMM was implemented across all NHS England acute mental health services, this could lead to approximately £69 million in cost savings, assuming 90% average occupancy, of which around a half would be cash-releasing. Full breakdowns of these costs and calculations are available in Table 10.

Buckley et al. [41] calculated that implementing VBPMM on an average-sized older adult inpatient mental health ward in England would result in cost savings of £413,651 (NHS & PSS) or £265,376 (NHS mental health trust) per year. This equates to a ROI of 14.04 (NHS & PSS) or 9 (NHS mental health trust). These calculations are based on estimates that a VBPMM system would cost £29,472 per ward per year and that introducing VBPMM would reduce costs per ward per year by £29,969 for night-time observations, £107,101 for one-to-one observations (cash releasing), £140,335 for one-to-one observations (opportunity cost saving), £142,948 (NHS & PSS) or £17,443 (NHS mental health trust) for bedroom falls at night, £18,308 (NHS & PSS) or £0 (NHS mental health trust) for A&E visits resulting from bedroom falls at night, and £4461 (NHS & PSS) for emergency services visits resulting from bedroom falls at night. A reduction in one-to-one observations was again identified as the primary driver of cost savings. They calculated that if VBPMM was implemented across all NHS England older adult mental health services, this could lead to cost savings of approximately £89 million, of which one-third would be cash-releasing. Full breakdowns of these costs and calculations are available in Table 10.

##### Complaints and damage

One study reported on VBPMM’s effect on complaints [47]; it reported that during the study period, no incidents related to VBPMM were recorded on the Trust’s online incident reporting system. During the first four nights of the new observation protocol (where VBPMM was used to conduct most observations of patients at night, instead of physical checks), eleven patients who completed questionnaires each night expressed no negative comments about the system. Details were not provided about how these patients were selected, or the format or content of the questionnaire.

#### Closed circuit television (CCTV)/video monitoring

Three studies [61, 66, 67] reported the effect of CCTV. One was rated high quality [61], one medium quality [67] and one low quality [66]. All three reported no conflicts of interest. One study had a cross-sectional design [61], one was mixed methods with a cross-sectional quantitative component [67] and one was a pre-post evaluation [66].

##### Violence and aggression

Two studies reported on CCTV’s effect on violence and aggression [66, 67]. It is unclear whether Warr et al. [67], who investigated the impact of CCTV use in patients’ bedrooms at night on the frequency and nature of incidents, conducted any statistical significance testing. However, they reported that there were fewer incidents at night compared to during the day, but that there was no difference in the nature of the incidents. They also stated that there was no evidence of any association between the nature of incidents and the presence or use of CCTV, or the choice of the patient to be observed using CCTV or not. Vartiainen & Hakola [66] did not conduct any statistical significance testing but reported that violent acts reduced on the CCTV-monitored wards.

##### Clinical outcomes

Two studies reported on CCTV’s effect on clinical outcomes [61, 66]. Simpson et al. [61] reported that CCTV (at exit) had no significant impact on substance or alcohol use on the ward. Vartiainen & Hakola [61] reported no significant changes in subjective mental health or paranoid traits, or ward atmosphere, on any of the wards (with or without CCTV).

##### Complaints and damage

One study reported on the impact of CCTV on damages [66]; it reported that no damage had occurred to cameras in two years of TV monitoring.

#### Body-worn cameras (BWCs)

Three studies reported the effect of BWCs [44, 52, 55]. All were rated low quality and one declared a conflict of interest [44]. One had a quasi-experimental repeated-measures pilot study design [44], one had a mixed methods uncontrolled pre-post pilot study design [55] and the other was a mixed methods repeated measures study [52].

##### Self-harm

One study examined the effect of BWCs on self-harm [52]. It reported no significant differences in self-harm before, during and after the BWC pilot phase on the acute ward, but significantly higher self-harm during the BWC trial period compared to pre- and post-trial periods on the PICU [52].

##### Violence and aggression

All three studies reported mixed results on the impact of BWCs on violence and aggression [44, 52, 55]. Ellis et al. [44] reported no significant changes in the overall numbers of violent and aggressive incidents. They reported a significant reduction in incident seriousness on two of the wards (“local services admissions” wards) but no significant changes on the remaining five wards. Hardy et al. [55] did not conduct statistical significance testing but stated that violence decreased on three wards and increased on two wards. They also noted an increase in verbal abuse on three wards, a decrease on one, and no change on another.

Foye et al. [52] found no significant differences in types of incidents (violence against people, violence against objects, verbal aggression, self-harm or conflict) before, during and after implementing BWCS on the acute ward. However, on the PICU, verbal aggression was significantly higher in the post-trial period compared to the pre-trial and trial periods but there were no significant differences in violence against people, violence against objects or conflict behaviour. They also reported no significant differences in incident severity or police involvement on the PICU across the trial periods, but on the acute ward, incident severity was significantly higher during the BWC trial period and post-trail periods compared to the pre-trial period, whilst police involvement was significantly lower in the post-trial period compared to the pre-trial and trial periods [52].

##### Restrictive practices

All three studies reported on restrictive practices [44, 52, 55]. Ellis et al. [44] reported no significant change in levels of incidents requiring restraint or rapid tranquillisation overall across the wards. They did report a significant decrease in rapid tranquillisation on the two local services admissions wards, but not on the five remaining wards. Hardy et al. [55] did not conduct significance testing but reported an increase in low-level restraint on two wards, a decrease on two, and no change on one. Hardy et al. [55] also noted a reduction in emergency restraint on three wards and an increase on two. Foye et al. [52] reported significant reduction in the use of restrictive practices on the acute ward in the post-BWC trial period compared to the pre-trial and trial periods, and a significant decrease in restrictive practices during the trial and post-trial periods on the PICU.

##### Complaints and damage

One study reported on BWCs’ effect on complaints and damage [55]. No statistical significance testing was conducted but they reported that three complaints were made during the BWC pilot period, none of which were related to a particular incident or restraint. They stated that this was lower than in the comparison period the previous year before BWC implementation, where eight complaints were made, two of which had related to an instance of restraint.

#### Global Positioning System (GPS) electronic monitoring

Two studies reported the effect of GPS electronic monitoring technology [56, 65]. Neither reported any conflicts of interest. One was rated as medium quality [56] and one low quality [65]. Both had date-matched pre-post study designs.

##### Absconding, leave violations, and leave type

Both studies reported on absconding and leave violations with GPS electronic monitoring [56, 65]. Tully et al. [65] reported that following the introduction of GPS monitoring, leave episodes were significantly more likely to be unescorted. There was no significant change in the odds of a leave episode resulting in leave violation during the initial follow-up (1 year later). However, during the subsequent follow-up (another year later), leave episodes were significantly less likely to lead to an incident of leave violation. Murphy et al. [56] reported no changes in the overall number of leave violations after implementing GPS electronic monitoring.

##### Complaints and damage

One study reported on complaints relating to GPS electronic monitoring [65]; it reported two events of patients challenging the use of GPS electronic monitoring. It did not report the number of patients involved and number of opportunities to challenge the use of GPS electronic monitoring.

##### Cost-effectiveness

One study reported on the cost-effectiveness of GPS electronic monitoring [65, 70]; it reported a no significant change in the average total cost per patient following the introduction of GPS electronic monitoring.

## Discussion

### Key findings

Our paper has summarised the use of surveillance technologies on inpatient wards internationally, how these technologies are being implemented, staff, patients’ and carers’ views and experiences of them, and the impact these technologies have on quantitative outcomes such as restraint, seclusion, self-harm, violence and aggression and absconding. There were no randomised controlled trials identified, and very few studies with control groups, meaning that causal inferences regarding the impacts of surveillance technologies cannot be drawn. Overall, there is currently insufficient evidence to suggest that surveillance technologies in inpatient mental health settings are achieving the outcomes they have been employed to achieve.

Key findings regarding implementation included a particular lack of research on certain types of surveillance technologies, such wearable sensors and GPS electronic monitoring, reflecting the novelty of these technologies in inpatient settings. Only two studies specified that they included wards with patients under the age of 18. There was more evidence of implementation of surveillance technologies in the UK than any other individual country. All of the studies on VBPMM and BWCs were UK-based, indicating an increasing adoption of these technologies in the UK [70]. All of the studies declaring conflicts of interest were examining these technologies, with 8/9 (88.9%) VBPMM studies and 1/6 (16.7%) BWC studies reporting conflicts of interest. Therefore, various factors may be driving research into VBPMM and BWCs in the UK, including national strategic directions promoting the integration of digital technologies in mental healthcare, pressures to explore potential healthcare cost-saving strategies, concerns about staff recruitment and retention, showcasing by regulatory bodies, and the financial interests of technology companies [71–75].

Our lived experience researchers highlighted discrepancies between the way surveillance technologies were described as being implemented in the literature and their use in practice. For example, they noted that in their experience, staff can decide to view multiple segments of VBPMM video feed instead of it only being viewable when vital sign measurements are made. This underlines the fact that this review only captures how surveillance technologies are described as being implemented in the included papers, and so does not capture the variety of ways in which they may be implemented in practice. Furthermore, it is important to consider that the implementation of surveillance technologies is dynamic, varying across contexts and evolving over time in response to technological innovations and developments in policies and practices.

There have been numerous efforts to develop an understanding of what “best practice” could look like given these technologies are already being implemented. Such guidelines have been established by healthcare regulatory bodies, professional associations and charities, as well as internal protocols by specific healthcare providers. This includes guidance around the use of surveillance technologies in general [76–78], as well as guidelines and recommendations for specific technologies such as BWCs [12], VBPMM [79, 80] and CCTV [81–83]. Given the growing use of differing surveillance techniques, further research to explore these guidelines and understand their commonalities and differences (e.g. how best practice may differ across cultures and countries) could provide a better position for developing a more robust message to those institutions implementing them.

Evidence from the included papers related to experiences should inform how best practice guidance is developed. Prominent themes in qualitative results were patients’ and staff’s ethical concerns about privacy invasion, data protection, patient confidentiality and informed consent, in-line with previous literature [15–17, 84]. These were reinforced by some quantitative evidence indicating that a substantial proportion of patients did not consent to the use of VBPMM [45] or understand the reasons for being monitored via video [38]. Only two studies specified that they included wards with patients under the age of 18; therefore, the literature fails to account for the unique ethical considerations when using surveillance technologies within children and young people’s care settings. These findings highlight the danger of surveillance technology use infringing upon patients’ human rights, choice and autonomy. If surveillance technologies are to be implemented in inpatient settings, establishing best practice guidance could potentially help to regulate their use and mitigate some of these adverse effects. However, additional oversight by regulatory bodies to ensure audits of standards and adherence would be required as simply developing and implementing best practice guidelines, standards and policies does not necessarily mean that they will be adhered to in practice. This was exemplified by Warr et al. [67] who highlighted instances in their study where patients were subject to surveillance via CCTV against protocol, at times it was not meant to be in use and with patients who had not consented to its use. Similar concerns are being articulated in lived experience literature [31].

Staff, patient and carer experiences of and attitudes towards surveillance technologies on inpatient wards in the included papers were complex, with variation both within and between these groups. This mirrors findings elsewhere on surveillance technologies [16, 17, 85]. Qualitative literature in this review revealed some perceptions that surveillance technology could reduce violence, aggression and self-harm in inpatient settings. However, quantitative papers examining these outcomes presented inconsistent or weak results. This finding is consistent with previous systematic reviews reporting a poor and inconsistent evidence base for the use of BWCs in public sector services, including law enforcement, physical and mental healthcare settings [14, 20, 86–88]. This dissonance between qualitative perceptions of surveillance technology in inpatient settings and quantitative evidence is noteworthy; it is unclear whether it is a result of poor-quality evidence, the limitations of the surveillance methods being employed or the complexity of the issues being addressed through surveillance and the context within which such endeavours take place. It is important to consider that perceptions of surveillance technologies are influenced not only by their effectiveness in practice, but also other external factors. These include, for example, how they are marketed by technology companies and described to people by staff, and broader societal attitudes towards surveillance, particularly amongst those more vulnerable and sensitive to close observation.

A notable discrepancy between the stated aims and the evidence base lies in assertions that surveillance technologies reduce costs [89]. Only four studies in this review explored the cost-effectiveness of surveillance technologies. One found that GPS electronic monitoring use in a forensic inpatient setting did not significantly decrease costs [56], whilst the other three reported potential cost savings associated with VBPMM use in PICU settings [42], acute mental health ward settings [41, 43] and older adult inpatient mental health services [41]. These economic analyses had notable limitations, such as most of the parameter values in the models being based on data from small numbers of wards within single services or NHS Trusts, and the models not considering costs such as maintenance, upgrades, wear and tear, ongoing staff training and data compliance administration. Downstream costs incurred from the impact of surveillance technologies upon outcomes such as length of inpatient stay, readmission rates and post-discharge service use were also not accounted for. Furthermore, the accuracy of some of the estimates used in the models has been called into question. For example, the economic models used in two studies [41, 43] assume VBPMM reduces self-harm in patient bedrooms by 44%. This may be an over-estimation because, while the only published study investigating VBPMM’s impact on self-harm reported a 44% *relative* reduction in self-harm rates in patients' bedrooms on two VBPMM wards compared to two control wards without VBPMM, the *actual* reduction in self-harm rates on the VBPMM wards alone was only 22% [45]. Additionally, these models calculated Accident and Emergency self-harm treatment costs using the weighted average of fracture codes, which risks over-estimating cost savings. Given these methodological limitations, the full ongoing costs of implementing surveillance technologies in inpatient mental health settings remains unknown, meaning that claims about their cost-effectiveness are not currently robustly substantiated by the evidence base.

In the three studies examining the cost-effectiveness of VBPMM, the main driver of identified potential cost savings was a reduction in one-to-one staff observations [41–43]. Qualitative evidence suggested that both staff and patients agreed that surveillance technologies should not replace or reduce human interaction. Indeed, research suggests that human contact, trust, support and empowerment form integral elements of therapeutic inpatient care, including during episodes of containment such as seclusion and restraint [15, 18]. Malcolm et al. [43] argue that a reduction in one-to-one staff observations with VBPMM could potentially free-up resources which could be used on other, more therapeutically beneficial activities. However, in practice, there is no guarantee that this freed-up staff time will be used for these purposes, posing a risk of reducing therapeutic interactions between staff and patients [90]. Over-reliance on technology and increased staff workloads (e.g. due to additional documentation requirements or inappropriate use of technology to justify assigning more patients to each member of frontline staff) could also lead to less vigilant and proactive care. As a result, surveillance technologies could promote more passive practice, where staff observe patients from a distance instead of engaging in therapeutic interactions with them. There is therefore a risk that the use of surveillance technologies to reduce staffing costs could result in decreased human interaction and so quality of care in inpatient settings.

Qualitative findings revealed that staff, patient and carer perceptions and experiences were mixed across the surveillance technology types. Some of the perceived benefits of surveillance technologies included improved staff and patient safety, enhanced monitoring and prevention of incidents (e.g. absconding, self-harm, violence and aggression) and facilitation of less intrusive observations of patients. Providing evidence to help investigate incidents and complaints was another perceived benefit, although some noted that surveillance technologies do not necessarily capture the entirety of events (e.g. due to some being turned on and off at the discretion of staff, and because they may not capture all of the events leading up to an incident). Concerns were also expressed by staff and patients that surveillance technology use could have wide-ranging negative effects, including negatively impacting patients’ recovery, privacy and dignity, decreasing feelings of safety, exacerbating distress and paranoia, reducing quality of care, damaging therapeutic relationships with staff and exacerbating power imbalances between patients and staff. Indeed, patient and service user groups, along with advocates and disability activists, have consistently voiced concerns about the potential iatrogenic harms associated with the use of surveillance technology in inpatient mental health settings [29, 91]. These harms have been the subject of media attention [29, 92], including news reports that at a recent inquest, concerns were raised about “alert fatigue” associated with surveillance technology use, suggesting that it can even have fatal consequences [93].

However, many of the included studies did not comprehensively investigate potential impacts, including unintended consequences, quantitatively. For example, very few quantitatively investigated surveillance technology’s impact upon patients’ mental health, absconding rates, self-harm or care quality. Further, even when these outcomes were investigated, there may have been limitations in how they were measured. For example, Ndebele et al. [45] only measured self-harm frequency in bedrooms and bathrooms, and so they did not capture any possible impact of VBPMM on rates of self-harm in communal ward spaces or on self-harm severity. This is a concern, given reports from patients that VBPMM use can worsen self-harm [31]. Many possible effects were not investigated at all in any of the included studies, such as the impact of surveillance technologies on therapeutic alliances, treatment satisfaction, staff and patient well-being, patient quality of life, recovery, engagement with services and longer-term outcomes such as readmission rates and post-discharge mental health and service use. Therefore, this review shows that our understanding of the impact of surveillance technologies in inpatient mental health settings, including their full range of potential harms and risks, remains incomplete.

#### Methodological quality of the included studies

There were significant methodological limitations in half (50.0%) of the included studies. Furthermore, there were declared conflicts of interest in over a quarter of studies (28.1%), all in studies examining VBPMM and BWCs. We also identified an additional potential undisclosed conflict of interest, and another VBPMM paper which was retracted from an academic journal due to an undeclared conflict of interest by the authors [94]. We noted that several of the studies with positive findings had declared conflicts of interest relating to the technology of interest, for example, studies being funded or conducted by the technology company themselves. This may not be surprising given their drive to demonstrate the efficacy of their technology. Many of these studies were also rated as low quality. Results therefore need to be interpreted with caution.

There was often a lack of information about how participants were recruited, and how surveys and interviews or focus groups were conducted, making it difficult to assess potential biases (e.g. risk of cherry-picking participants, excluding the most unwell patients, power imbalances inhibiting sharing of criticisms of technology by patients and staff). Consequently, the literature may underrepresent the perspectives of populations facing greater barriers to research participation (e.g. patients lacking capacity to consent, people with concerns about confidentiality, distrust towards research or facing language barriers). The lack of transparency in methodologies, e.g. no pre-registration of studies, makes it difficult to ascertain how reported outcomes were chosen, and raises questions around whether negative outcomes (such as harms, verbal aggression and property destruction) were purposefully omitted. Methodologically, no randomised controlled trials were identified, and few studies had control groups, with mainly before and after comparisons. Many papers did not adequately consider the complexity of the issues and variables surrounding surveillance, for instance, the role of confounding or contextual factors in interpreting results.

There was in general a significant lack of lived experience involvement in the implementation and evaluation of surveillance technologies, and a lack of lived experience involvement in the studies themselves. Even when it was reported, it was often poorly described, for example, lacking detail about numbers of people involved, their demographics, recruitment methods and how (and to what degree) they were involved in the research process. Furthermore, in some studies there lacked a clear distinction between the involvement of individuals with lived experience in the research process versus participation in the study by patients.

#### Strengths and limitations

Our review is a comprehensive, systematic synthesis of the available literature on the implementation, experience and impact of surveillance technologies in inpatient mental health settings. We reported information on lived experience involvement in the study design and the implementation of the surveillance, exposing significant gaps which should be addressed and prioritised. We also reported information on declared conflicts of interest and funding in the included papers, which have enhanced our ability to assess the validity and independence of the evidence presented.

We sought to identify both academic and grey literature in our review, although, due to time constraints, grey literature was only included in relation to research objective 2 (exploring patient, staff and carer perceptions and experiences of the technology) and was limited to studies which included a description of their methodology. We acknowledge that there may be perspectives which are therefore underrepresented in our synthesis, including perspectives from those with lived experience of surveillance on inpatient wards. Another limitation is that we did not contact authors of included studies for missing information. Had this information been obtained, it may have impacted our quality appraisal ratings. There is a risk of publication bias (i.e. studies showing positive outcomes being more likely to be published) given the number of included studies which declared conflicts of interest, although we were unable to investigate and confirm this.

#### Implications for policy and practice

The findings of this review suggest that the current evidence base does not support the use of surveillance technologies as a means of improving safety, care quality or reducing costs in inpatient mental health settings.

More independent, coproduced research is needed thoroughly evaluating the impact of surveillance technologies, including their full range of potential harms, in inpatient settings. As is best practice with the implementation of any new intervention, they should only be deployed if the resulting evidence supports their use.

However, the current reality is that surveillance technologies are already being implemented across a variety of inpatient services across the globe, and it is unlikely that this will come to a complete halt. If these technologies continue to be implemented, there will be an urgent need to develop trauma-informed policies, procedures and guidelines for their use, centred around the perspectives of patients. This could contribute to developing more acceptable ways of using surveillance technologies and help maximise their potential benefits and mitigate their harms. These guidelines and policies would need to be accompanied by comprehensive and ongoing training for staff, ideally coproduced with patients, and systematic monitoring and auditing of services’ adherence to them to help ensure compliance.

These policies and guidelines should comprehensively address the tensions and ethical concerns highlighted by patients, carers and staff in this review. This includes concerns around informed consent, patient confidentiality, data protection and potential iatrogenic harms. They should also include procedures for informing patients about the use of surveillance technologies, routinely monitoring and discussing their usage, and investigating and addressing misuse of technology and data. Consideration should also be given to measures to protect patients’ dignity and privacy and prevent (re)traumatisation, for example, pixelating or blurring footage of sensitive areas of patients’ bodies when using video monitoring technologies. Wider systemic challenges, including issues such as staffing shortages, power imbalances and reliance on restrictive approaches to risk management, also need to be acknowledged and actively addressed.

It is essential that all stakeholders, particularly patients, are meaningfully involved in all stages of future research, implementation, evaluation and decision-making regarding surveillance technology use in inpatient mental health settings.

#### Implications for research

The literature base identified in this review is largely characterised by uncontrolled and poor-quality studies presenting inconsistent results. Over a quarter of papers identified in this review had declared conflicts of interest, and an additional potential undisclosed conflict of interest was also identified.

Future research on surveillance in inpatient wards should be funded and conducted independently to ensure the rigour and validity of the methods and findings. Conflicts of interest should also be declared in any published reports or articles. Research led by those with lived experience of mental health inpatient care generally, and surveillance technologies specifically, would be particularly valuable in evaluating potential harms missed by academic or clinical researchers. Care should also be taken to ensure that the perspectives of those who are unwell, or may need support to express their views, are captured in any future research on technologies in these settings [95]. Further synthesis of data on surveillance from other locations where people with mental health problems present may be helpful, for example in crisis services or mental health presentations in emergency departments.

Future primary research in this area should more purposefully aim to (i) investigate the harms caused by surveillance, including a full exploration of the psychological impact and an exploration of changes in care protocols due to the technology, (ii) explore and establish best practice and ethical guidelines for the use of surveillance in inpatient units (and across all mental health services and settings) which fully consider the experiences of patients who have negative views and adverse responses to surveillance, and (iii) include those with lived experience in study design, analysis, interpretation and dissemination.

## Conclusions

This systematic review has found insufficient evidence to suggest that surveillance technologies in inpatient mental health settings are achieving their intended outcomes, such as improving safety, enhancing care quality and reducing costs. Qualitative findings indicate that patient, staff and carer experiences of surveillance technologies are mixed and complex. Whilst some people perceived potential benefits, such as reductions in incidents, less intrusive monitoring and provision of evidence to aid investigations, others raised concerns about their potential harms, including ethical concerns about their impact on patients’ human rights, privacy, dignity, and recovery. The reviewed studies were generally of low methodological quality, lacked lived experience involvement, and a considerable proportion disclosed conflicts of interest. Further independent, co-produced research is needed to more comprehensively evaluate the impact of surveillance technologies in inpatient settings, including their full range of potential benefits and harms.

## Lived experience commentary, by Georgia Johnson and Rachel Rowan Olive

We are unsurprised by the poor quality and inconsistent results of the evidence. In our experience surveillance technology—like most restrictive practice—is rapidly rolled out in response to institutional anxiety following serious incidents. Surveillance technology’s illusion of control alleviates that anxiety, promising potential benefits well beyond the evidence base. Surveillance’s damage, however, is more concrete. Most researchers did not look for iatrogenic harm, thus compounding said harm by invalidating our fears and experiences.

But we know these harms intimately, because we have experienced them. These digital technologies strip away our most basic dignity, and are, by an extension, an affront to our very humanity. It is when professionals stop treating us like humans, and see only a cluster of symptoms, that restrictive practice becomes its most abusive self. Other people’s fear is not a justification for abusing us in this way.

The UK’s psychiatric system is not one where meaningful consent for surveillance can be implemented; however, blithely manufacturers and evaluators state that consent is always obtained. When Oxevision was piloted on Georgia’s ward, she was not given the opportunity to consent: she only discovered the system existed after a nurse said, “Oh you’re in the bathroom, I couldn’t see you on the camera.” Staff didn’t know whether patients were allowed to refuse it. The distress caused was so great that the response team had to be called. After turning the cameras off, they were turned back on during another shift. When Georgia objected, staff said that no such cameras existed and that she was experiencing psychosis. *Would she like a cup of tea instead?* FOI data from StopOxevision [96] shows this is not an isolated event, with patient leaflets and posters frequently omitting any mention of functionalities such as camera surveillance.

Finally, we highlight the contrast in attitudes to staff surveilling patients versus patients filming staff. On being illegally detained during a mental health crisis, Rachel began recording those detaining her, knowing we are frequently disbelieved when making complaints. Outraged staff *wearing body-worn cameras* promptly insisted, “we are not here to be filmed”.

This is a common response to patients documenting poor experiences; it puts paid to any illusion that institutional surveillance could lessen the violent disbelief we face. Staff control when and how cameras are used. Surveillance within this system only cements power imbalances and causes lasting trauma.

## Supplementary Information

Additional file 1: Appendices. Appendices A-F include the PRISMA checklist, explanations of protocol deviations, full search strategies, excluded full texts and their reasons for exclusion, a detailed study characteristics table, and detailed table summarising the implementation of surveillance technologies in the included studies.

Additional file 2. Data extraction form template. Data extraction form template used to extract data from the included studies in this review.

Additional file 3. Mixed Methods Appraisal Tool quality ratings. Excel spreadsheet showing the ratings for each domain of the Mixed Methods Appraisal tool for each of the included studies.

## Data Availability

The template data extraction form is available in Additional File 2. MMAT quality appraisal ratings for each included study are available in Additional File 3. All data used is publicly available in the published papers included in this study.

## Abbreviations

BWCs: Body-worn cameras
CCTV: Closed circuit television
CI: Confidence interval
GPS: Global Positioning System
IT: Information Technology
MHPRU: Policy Research Unit in Mental Health
MMAT: Mixed Methods Appraisal Tool
NHS: National Health Service
NIHR: National Institute for Health and Care Research
PANSS: Positive and Negative Syndrome Scale
PICU: Psychiatric intensive care unit
PIN: Personal Identification Number
PMVA: Prevention and Management of Violence and Aggression
PRISMA: Preferred Reporting Items for Systematic Reviews and Meta-Analyses
PSS: Personal Social Services
ROI: Return on investment
SD: Standard deviation
TV: Television
UK: United Kingdom
USA: United States of America
VBPMM: Vision-Based Patient Monitoring and Management
