# Correction: The use and impact of surveillance-based technology initiatives in inpatient and acute mental health settings: a systematic review

**DOI:** 10.1186/s12916-025-03979-2

**Published:** 2025-03-08

**Authors:** Jessica L. Griffiths, Katherine R. K. Saunders, Una Foye, Anna Greenburgh, Ciara Regan, Ruth E. Cooper, Rose Powell, Ellen Thomas, Geoff Brennan, Antonio Rojas-García, Brynmor Lloyd-Evans, Sonia Johnson, Alan Simpson

**Affiliations:** 1 Department of Health Service and Population Research (HSPR), NIHR Policy Research Unit in Mental Health (MHPRU), Institute of Psychiatry, Psychology & Neuroscience, King’s College London, London, UK; 2 NIHR Policy Research Unit in Mental Health (MHPRU), Division of Psychiatry, University College London, London, UK; 3 Department of Health Service and Population Research (HSPR), Institute of Psychiatry, Psychology & Neuroscience, King’s College London, London, UK; 4 Florence Nightingale Faculty of Nursing, Midwifery and Palliative Care, King’s College London, London, UK; 5 School of Geography, Politics, and Sociology, Newcastle University, Newcastle Upon Tyne, UK; 6 London, UK; 7 Department of Behavioural Science Methodology, Faculty of Psychology, University of Granada, Granada, Spain; 8 Camden and Islington NHS Foundation Trust, London, UK

**Correction** **:** **BMC Medicine 22, 564 (2024)**

**https://doi.org/10.1186/s12916-024-03673-9**

The authors wish to note an amendment to the following text in the original article:

‘[…] This may be an over-estimation because, while the only published study investigating VBPMM’s impact on self-harm reported a 44% relative reduction in self-harm rates in patients' bedrooms on two VBPMM wards compared to two control wards without VBPMM, the actual reduction in self-harm rates on the VBPMM wards alone was only 22% [1]. Additionally, these models calculated Accident and Emergency self-harm treatment costs using the weighted average of fracture codes, which risks over-estimating cost savings […]’

The authors wish to acknowledge the ‘Stop Oxevision’ campaign’s contribution in the context of this text, and to note that it should instead read as follows:

‘[…] The Stop Oxevision campaign [2] argues that this may be an over-estimation because, while the only published study investigating VBPMM’s impact on self-harm reported a 44% relative reduction in self-harm rates in patients’ bedrooms on two VBPMM wards compared to two control wards without VBPMM, the actual reduction in self-harm rates on the VBPMM wards alone was only 22% [1, 3]. Additionally, they note that these models calculated Accident and Emergency self-harm treatment costs using the weighted average of fracture codes, which risks over-estimating cost savings [3] […]’
